# Phosphorylated Ribosomal Protein S6 Is Required for Akt-Driven Hyperplasia and Malignant Transformation, but Not for Hypertrophy, Aneuploidy and Hyperfunction of Pancreatic β-Cells

**DOI:** 10.1371/journal.pone.0149995

**Published:** 2016-02-26

**Authors:** Avigail Dreazen Wittenberg, Shahar Azar, Agnes Klochendler, Miri Stolovich-Rain, Shlomit Avraham, Lea Birnbaum, Adi Binder Gallimidi, Maximiliano Katz, Yuval Dor, Oded Meyuhas

**Affiliations:** 1 Department of Biochemistry and Molecular Biology, The Institute for Medical Research–Israel-Canada, The Hebrew University-Hadassah Medical School, Jerusalem, Israel; 2 Department of Developmental Biology and Cancer Research, The Institute for Medical Research–Israel-Canada, The Hebrew University-Hadassah Medical School, Jerusalem, Israel; University of Bremen, GERMANY

## Abstract

Constitutive expression of active Akt (Akt^tg^) drives hyperplasia and hypertrophy of pancreatic β-cells, concomitantly with increased insulin secretion and improved glucose tolerance, and at a later stage the development of insulinoma. To determine which functions of Akt are mediated by ribosomal protein S6 (rpS6), an Akt effector, we generated mice that express constitutive Akt in β-cells in the background of unphosphorylatable ribosomal protein S6 (rpS6^P-/-^). rpS6 phosphorylation deficiency failed to block Akt^tg^-induced hypertrophy and aneuploidy in β-cells, as well as the improved glucose homeostasis, indicating that Akt carries out these functions independently of rpS6 phosphorylation. In contrast, rpS6 phosphorylation deficiency efficiently restrained the reduction in nuclear localization of the cell cycle inhibitor p27, as well as the development of Akt^tg^-driven hyperplasia and tumor formation in β-cells. *In vitro* experiments with Akt^tg^ and rpS6^P-/-^;Akt^tg^ fibroblasts demonstrated that rpS6 phosphorylation deficiency leads to reduced translation fidelity, which might underlie its anti-tumorigenic effect in the pancreas. However, the role of translation infidelity in tumor suppression cannot simply be inferred from this heterologous experimental model, as rpS6 phosphorylation deficiency unexpectedly elevated the resistance of Akt^tg^ fibroblasts to proteotoxic, genotoxic as well as autophagic stresses. In contrast, rpS6^P-/-^ fibroblasts exhibited a higher sensitivity to these stresses upon constitutive expression of oncogenic Kras. The latter result provides a possible mechanistic explanation for the ability of rpS6 phosphorylation deficiency to enhance DNA damage and protect mice from Kras-induced neoplastic transformation in the exocrine pancreas. We propose that Akt1 and Kras exert their oncogenic properties through distinct mechanisms, even though both show addiction to rpS6 phosphorylation.

## Introduction

Pancreatic β-cell mass is a prime determinant of glucose homeostasis and is regulated by a dynamic balance of proliferation, cell size, apoptosis and neogenesis [1], involving both mitogenic and growth signals. These signals are initiated by activation of growth factor receptor tyrosine kinases, which in turn lead to activation of phosphatidylinositol 3-kinase (PI3K). PI3K converts the lipid phosphatidylinositol-4,5-P2 (PIP2) into phosphatidylinositol-3,4,5-P3 (PIP3), in a reaction that can be reversed by the PIP3 phosphatase PTEN (phosphatase and *tensin* homolog deleted from chromosome 10) [2]. PIP3 recruits both 3-phosphoinositide-dependent kinase 1 (PDK1) and Akt to the plasma membrane [3], and PDK1 phosphorylates and activates Akt [4].

There are three closely related isoforms of Akt in mammalian cells, Akt1, Akt2 and Akt3 [5]. Mice, whose β-cells overexpress a constitutively active Akt1 (Akt^tg^) bearing a myristoylation signal (myr-Akt), display a prominent increase in both the number and size of these cells, concomitantly with improved glucose tolerance [6, 7]. Likewise, conditional activation of Akt in β-cells results in fasting hypoglycemia, hyperinsulinemia and improved glucose tolerance [8]. Akt exerts these effects by phosphorylating tuberous sclerosis complex 2 (TSC2) and thereby inhibiting the ability of TSC1-TSC2 complex to act as a GTPase-activating protein (GAP) for Rheb (Ras-homolog enriched in brain). Consequently, the mammalian target of rapamycin (mTOR) complex 1 (mTORC1) is derepressed. Indeed, mice with conditional deletion of Tsc2 in β-cells exhibit lower glucose levels, hyperinsulinemia and improved glucose tolerance. These changes are explained by increases in β-cell mass, proliferation and cell size [9]. The role of mTORC1 as a transducer of some Akt signals is demonstrated by the ability of rapamycin, an mTORC1 inhibitor, to abolish the Akt1-induced β-cell proliferation [10].

Once mTORC1 is activated it regulates protein synthesis by direct phosphorylation of (a) eukaryotic initiation factor (eIF) 4E-binding proteins (4E-BP1, 2 and 3), which consequently dissociate from and derepress eIF4E; and (b) ribosomal protein S6 kinases (S6K1 and 2) which become fully active and affect the protein synthesis machinery (reviewed in [11]). Consistently, mice deficient of S6K1 display glucose intolerance, hypoinsulinemia and reduced β-cell size [12], whereas mice over expressing a constitutively active form of S6K in β-cells display increased insulin secretion in the absence of changes in β-cell mass [13].

Ribosomal protein S6 is the best-characterized substrate of S6K [14]. A knockin mouse (rpS6^P-/-^), in which all five phosphorylatable serine residues of rpS6 were substituted by alanines, displays a small size phenotype that reflects a cell type-specific growth defect in both pancreatic β-cells and myotubes [15, 16]. Moreover, rpS6^P-/-^ mice have diminished levels of pancreatic insulin, hypoinsulinemia, impaired glucose tolerance, reduced muscle energy content, compromised compensatory renal hypertrophy and impaired parathyroid hormone secretion after experimental uremia [15–18]. Interestingly, we have recently shown that rpS6 phosphorylation deficiency enhances Kras-induced DNA damage in the exocrine pancreas and consequently boosts p53-mediated tumor suppression [19].

Activation of Akt1 has been detected in approximately half of pancreatic ductal adenocarcinoma cancer patients [20], and it appears to exert its oncogenic activity in this cancer by overcoming cell cycle arrest, [21, 22] blocking apoptosis [23, 24], and promoting angiogenesis [25]. The involvement of Akt in carcinogenesis is not confined to the exocrine pancreas, as mice expressing constitutively myr-Akt1 in their β-cells exhibit islet hyperplasia leading to insulinoma formation. This transformation event is mediated by S6K1 and is fully blocked in S6K1 deficient mice [26].

The parallel roles previously assigned for Akt and its downstream effector, rpS6 phosphorylation, as determinants of the size and function of β-cells, as well as of pancreatic tumorigenicity, prompted us to examine whether Akt exerts all these effects by signaling to rpS6 phosphorylation. Surprisingly, we found that Akt1 controls β-cell size in an rpS6 phosphorylation-independent fashion, yet the development of insulinomas in Akt^tg^ mice is abolished if their β-cells lack rpS6 phosphorylation. Mechanistically, rpS6 phosphorylation deficiency failed to protect β-cells from Akt^tg^ induced aneuploidy, and therefore, does not block insulinomas development by interfering with their ploidy. Contrarily, immortalized rpS6^P-/-^ MEFs exhibit impaired translation fidelity, even in the presence of Akt^tg^, potentially contributing to the antitumorigenic effect of rpS6 phosphorylation deficiency.

## Material and Methods

### Animals

**Fig 1:**
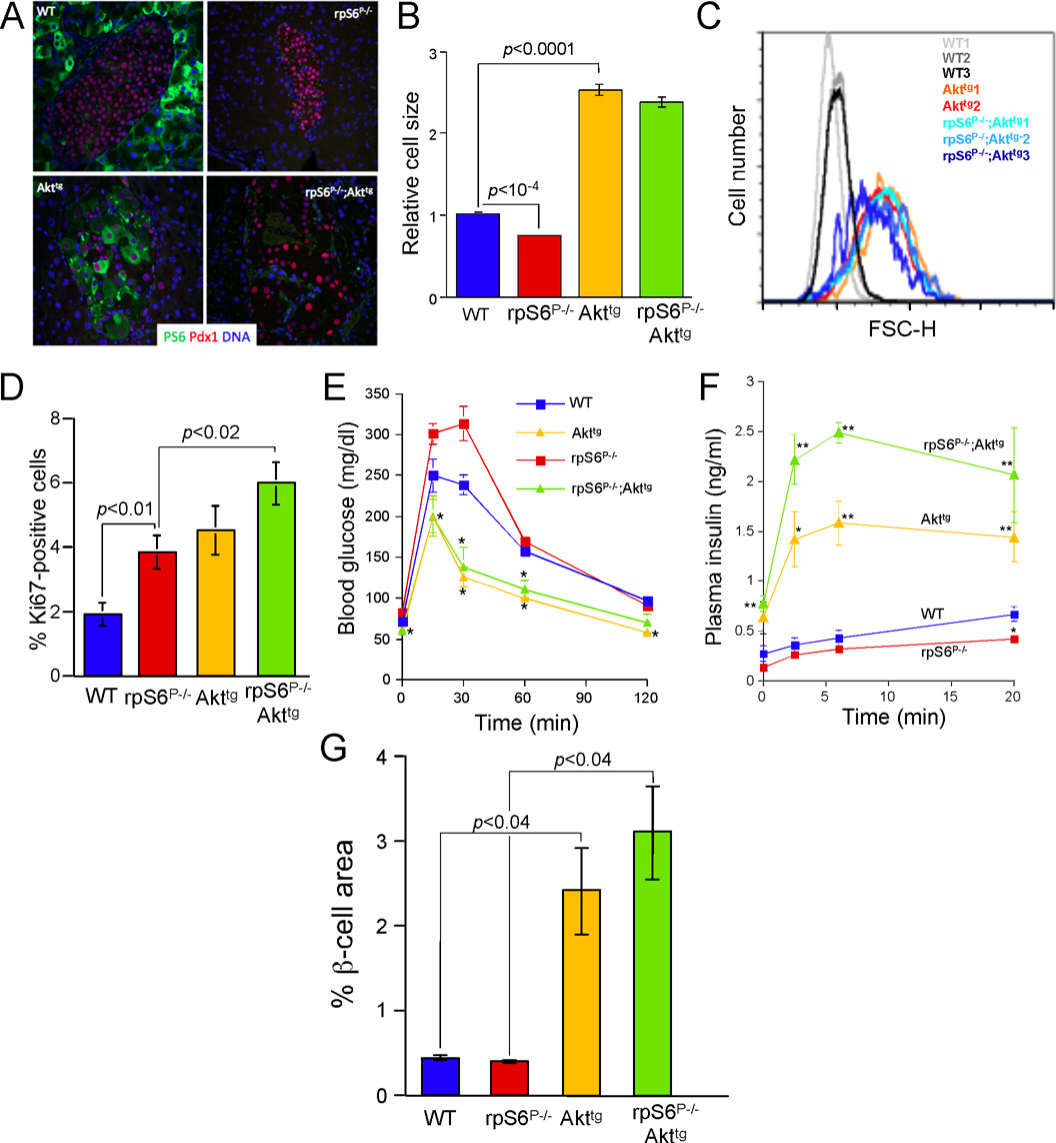
Akt overexpression overrides the inhibitory effect of rpS6 knockin on β-cell size and function. (A) Representative images of β-cells of all four genotypes. Pancreatic sections for WT, rpS6^P-/-^, Akt^tg^ and Akt^tg^; rpS6^P-/-^ 2-month-old mice were immunostained for phospho-rpS6 (green), for Pdx1 (red) and for DNA by Sytox (blue). All images are set to the same scale. (B) Pancreatic sections from 2-month-old mice were stained for DNA by Sytox and for β-cells cytoplasm by anti-insulin antibodies. The size of β-cells was derived from the reciprocal value of their density, which represents the number of nuclei in multiple 2,500-μm^2^ squares that contained only insulin-positive cells. The relative cell size was attained by normalizing to that of wild type mice, arbitrarily set at 1. Values are presented as a mean ± SEM of counts derived from 121 to 189 squares within 21 to 31 islets identified in 3 to 6 mice of each genotype. (C) β-cells were isolated from 2-month-old mice (three wild-type (WT), two Akt^tg^ and three rpS6^P-/-^;Akt^tg^ mice), fixed, permeabilized, immunostained for insulin, HA, Ki67 and Hoechst before FACS analysis. The cell size of quiescent β-cells (HA- and insulin-positive, Ki67-negative) was measured as described in Material and Methods. Notably, The percentage of HA-positives in isolated β-cells from 3 rpS6^P-/-^;Akt^tg^ mice was 78.3 ± 0.7 (n = 3) and from 2 Akt^tg^ mice was 66.5 (65% and 68%) (D) Pancreatic sections, from 2-mo-old mice of the indicated genotypes, were stained for DNA by Sytox green, for proliferation by anti-Ki67 and for β-cells cytoplasm by anti-insulin antibodies (see S2 Fig for representative islets of all 4 genotypes). The proliferation is presented as % Ki67 positive within insulin positive cells. Values are presented as a mean ± SEM of counts derived from 20 to 50 islets identified in 3 to 4 mice of each genotype. (E) Glucose tolerance test. Blood glucose concentrations before and after intraperitoneal injection of 2.5 g D-glucose per kg body weight in 6 to 10-weeks-old mice fasted for 17 h. The data represent an average ± SEM for at least 11 mice for each genotype. (*) p < 0.002 versus rpS6^P-/-^ mice. (F) Glucose-stimulated insulin secretion. Serum insulin concentrations before and after intraperitoneal injection of 2.5 g D-glucose per kg body weight in 6 to 10-weeks-old mice, which had been fasted for 17 h. The data represent an average ± SEM for at least 4 to 7 mice for each genotype. (*) p < 0.05; (**) p < 0.01. (G) The β-cell area is presented as a mean ± SEM of the percent of total pancreatic area in 3 mice of each of the indicated genotypes.

**Fig 2:**
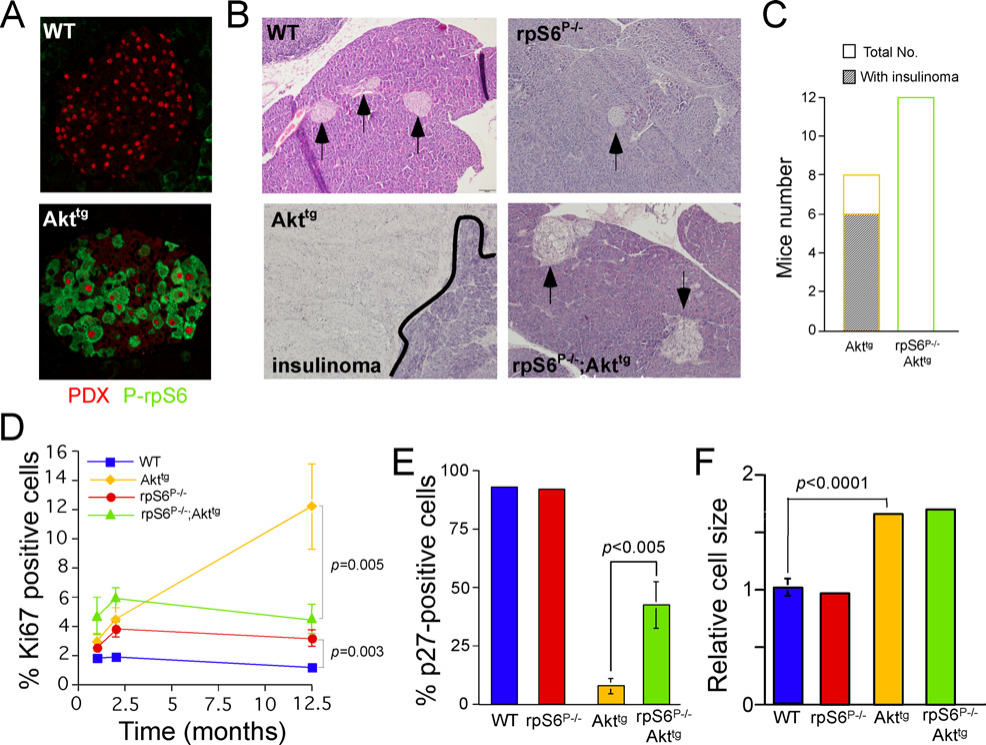
Rescue of rpS6 phosphorylation deficient mice from myr-Akt1-induced insulinoma is associated with decreased cell proliferation and increased p27 nuclear localization. (A) Pancreatic sections of islets from 12-month-old WT and Akt^tg^ mice were stained for phosphorylated rpS6 and (green) and for β-cells nuclei by PDX1 antibodies (red). (B) Pancreatic sections from WT, rpS6^P-/-^, Akt^tg^ and rpS6^P-/-^;Akt^tg^ 12-month-old mice were stained by haematoxylin-eosin. All images presented at the same magnification (see size bar [100 μm] in WT). Arrows point to islets and the black line (lower left panel) marks the right margin of the insulinoma. (C) The number of mice with readily detectable insulinoma (dashed-line box) is presented within the total number of examined Akt^tg^ and rpS6^P-/-^;Akt^tg^ mice (open box). (D) Pancreatic sections from1, 2 and 10 to 15-mo-old mice of the indicated genotypes were stained as described in the legend to Fig 1D (see S3 Fig for representative islets from all 4 genotypes). The proliferation is presented as % Ki67 positive within insulin-positive cells. Values are presented as a mean ± SEM of counts derived from 20 to 50 islets identified in 3 to 4 mice of each genotype at each time point. (E) Pancreatic sections from 10 to 15-mo-old mice were stained for p27 and for insulin (see S4 Fig for representative islets from all 4 genotypes). Values are presented as a mean ± SEM of counts derived from 12 to 42 islets identified in 2 to 4 mice of each genotype. (F) The size of β-cells in 10 to 15-mo-old mice was analyzed and calculated as described in the legend to Fig 1C. Values are presented as a mean ± SEM of counts derived from 215 to 1252 squares (2,500- μm^2^) within 21 to 31 islets identified in 2 to 7 mice of each genotype.

Generation of rpS6^P-/-^ knockin as well as RIP-MyrAkt1–expressing mice (*Akt1*^*tg*^*)* has been previously described [7, 15]. Both *rpS6*^*P-/-*^ and RIP-MyrAkt1 were on C57BL/6 genetic background. The C57BL/6 *rpS6*^*P-/-*^ mice were generated by backcrosses of *rpS6*^*P-/-*^ 129/svj with C57BL/6 Akt^tg^. The *rpS6*^*P-/-*^ and Akt^tg^ strains were crossed to obtain rpS6^P-/+^ and Akt^tg+/-^ mice, which were intercrossed to yield Akt^tg^ and rpS6^P-/-^;Akt^tg^, respectively. Mice were genotyped by PCR analysis using specific primers for myr-Akt: (5’ CAGGCAAGTGTTTGGAAACTGC 3’ and 5’ AAAGGTCTTCATGGTGGCACCGTC 3’) as well as for the rpS6^P-/-^ and rpS6^P+/+^ alleles [15]. Mouse embryo fibroblasts MEFs were prepared from embryos of S6^loxP/loxP^;CreER^+^ mice at day 13.5 of gestation as described previously [27]. Immortalized MEFs were obtained by transfection with a plasmid expressing the Simian Virus 40 large T-antigen. Mice were housed in plastic cages and maintained at 22°C with a 12-hour dark/12-hour light cycle and had free access to food and water. Notably, all experiments described in Figs 1 and 2 were carried out with female mice, since the establishment of the role of rpS6 phosphorylation as a determinant of glucose homeostasis had been originally conducted with female mice [15].

### Ethics Statement

The joint ethics committee (IACUC) of the Hebrew University and Hadassah Medical Center approved the study protocol for animal welfare. The Hebrew University is an AAALAC international accredited institute. Mice were euthanized by cervical dislocation after anesthesia with isoflurane. Animals were monitored weekly for body weight, blood glucose and general appearance. Mice that showed severe hypoglycemia (<50 mg/dL glucose) or lost >10% body weight were euthanized.

### Immunofluorescence Microscopy of Pancreas Sections

Five-micron thick sections from formalin-fixed, paraffin-embedded pancreases were prepared. Slides were rehydrated, blocked with CAS-block (Invitrogen) and incubated overnight at 4°C with primary antibodies. After washing in PBST slides were incubated for 1 h with fluorescent secondary antibodies, washed again, incubated briefly with Sytox Green Nucleic Acid Stain (Invitrogen) and mounted. Digital images of islets and acinar tissue were obtained using an Olympus BX51 microscope (200 X magnification), based on staining for insulin. The number of nuclei in a constant area (2500 μm^2^) was counted manually using Image-Pro Plus software (Media Cybernetics). The primary antibodies used were guinea pig anti-insulin (Dako), mouse anti-p27 (Santa Cruz), goat anti-PDX1 (a gift from Christopher VF Wright), rabbit anti-phospho rpS6(240/244) (Cell Signaling Technology, Beverly, MA, USA), and rabbit anti-Ki67 (Thermo Scientific). The secondary antibodies used were CY2-anti-guinea pig and CY3-anti rabbit (Jackson ImmunoResearch). Immunohistochemical staining of pancreatic sections for HA were carried out with mouse anti-HA (Cell Signaling Technology).

### Assessment of β-Cell Area

To assess β-cell area, consecutive paraffin sections, 75 μm apart, spanning the entire pancreas (approximately 9 sections/pancreas) were stained for insulin and hematoxylin. Digital images of the entire area of each section at a magnification of x4 were obtained and stitched using NIS-Elements software, and the fraction of tissue area covered by insulin staining was determined. The percentage β-cell area was defined as the β-cell area divided by pancreatic section area, multiplied by 100.

### Identification of Insulinomas

Blood glucose was monitored in aged Akt^tg^ mice (10 mo. and older) exhibiting distress signs. Mice with apparent hypoglycemia were sacrificed and their pancreata were subjected to gross pathological examination. Pancreatic masses at least 2 mm in size in hypoglycemic mice were defined as insulinomas.

### Glucose Tolerance Tests

A glucose tolerance test was performed on fasted (17 h) mice by injecting D-glucose intraperitoneally at a dose of 2.5 g/kg body weight. Whole venous blood was obtained from the tail at the indicated time points after the glucose load. Blood glucose levels were measured using a glucometer (Bayer).

### *In vivo* Insulin Secretion Tests

Fasted (17 h) mice were intraperitoneally injected with D-glucose at a dose of 2.5 g/kg body weight. Whole venous blood was obtained from the tail at the indicated time points after glucose load and serum insulin was measured by Ultrasensitive Insulin Mouse Elisa Kit (CrystalChem).

### Cells

Primary and immortalized (SV40 T-antigen transfected) rpS6^P+/+^ (WT) and rpS6^P-/-^ mouse embryonic fibroblasts (MEF) were prepared and maintained in culture as previously described [15]. HEK293 cells were grown in Dulbecco's modified Eagle's medium (DMEM) containing 10% fetal calf serum, 2 mM glutamine, 100 u/ml penicillin and 0.1 mg/ml streptomycin.

### Analysis of β-cell ploidy

Pancreatic islets were isolated and dissociated with trypsin prior to fixation and permeabilization with Fix/Perm and Perm/Wash solutions (BD Biosciences). Islet cells were then immunostained for insulin (Guinea Pig anti-insulin [Dako]) or HA (Mouse anti-HA-Tag [Cell Signaling Technology]) to mark β-cells from WT or Akt^tg^ mice, respectively, and for Ki67 to detect proliferating cells. Cells were incubated with 10 μg/ml Hoechst 33342 for 10 min. at room temperature. DNA content of quiescent (G0) β-cells (Ki67-negative) was profiled by flow cytometric analysis of 5,000 to 10,000, using FACSAria cytometer (BD Bioscences) equipped with a UV laser.

### Determination of Size of Isolated Islets Cells

The average cell size in 5,000–10,000-cell samples was assessed using the FACStarplus flow cytometer (Becton Dickinson) with Cell Quest software. The resulting parameter, mean forward scatter height (FSC-H), is a measure of relative cell size.

### Western Blot Analysis

Immunoblotting was performed as described [28], using antibodies against rpS6 (#2217), phospho rpS6(Ser240/244) [#2215], Akt [#9272], phospho Akt(ser473) [#4058], β-actin [#4967], α-tubulin [#2144], cleaved caspase 3 (Asp175) [#9661], LC3B [#2775], Ras [#3339] from Cell Signaling Technology, Beverly, MA, USA; and against puromycin (clone 12D10) from Millipore. All antibodies were diluted 1:1000.

### Cloning

Lentiviral vector expressing myristoylated, filled in 1224 bp *Sal*I-*Cla*I fragment containing constitutively active form of Akt (Myr-Akt [Δ4–129]-HA) was excised from pLNCX1 myr-Akt and ligated in between filled in *Not*I and *Bam*HI sites of pHAGE (a lentivirus core plasmid [29] yielding HAGE2-myr-Akt. Cloning of mouse rpS6 coding sequence with different residues replacing the phosphorylatable serine residues into pHAGE was carried out in multiple steps: a) generation of PCR fragments encoding rpS6 with 5 Serine [5S], 5 Alanine [5A], or 5 Aspartic acid [5D; b) cloning of these fragment into pcDNA3.1; c) a 800 bp PCR fragments generated using each of the pcDNA3.1 cloned rpS6 variants was digested with *Bgl*II and *Age*I and ligated in between *Bgl*II and *Age*I sites in pEGFP-N1 (Clontech) to yield GFP open reading frame (ORF) followed in frame by the carboxy terminus of the rpS6 variant. A 1500 bp filled in *Bgl*II-*Not*I fragment from each of these subclones was ligated in between filled in *Not*I and *Bam*HI sites in pHAGE to yield pS6^5S^-GFP, pS6^5A^-GFP and pS6^5D^-GFP.

### Virus Production and Infection

Generation of lentiviral vectors was accomplished by a five-plasmid transfection procedure [30]. Briefly, 293T cells were transfected using polyethylenimine (PEI) procedure with a pHAGE-derived backbone construct together with four expression vectors encoding the packaging Gag-Pol, Rev, Tat, and the G protein of the vesicular stomatitis virus (VSV-G) (10, 0.5, 0.5, 0.5, and 1 μg, respectively, per 100 mm plate) and 0.5 μg PEI. The mixture was added to the cells, maintained in the growth medium. In the next morning the medium was replaced with 5% FBS-containing medium for 4 h and then with 1% FBS-containing medium till the next morning. Medium was collected 48 h post transfection, spun down at 1500 rpm for 5 min, and the supernatant was passed through a 0.45- μm filter and kept at 4°C. 5 ml of the viral suspension were treated with 60 μg of polybrene (Sigma) at room temperature for 20 min. and then added to HEK293 cells in 100 mm plate. 4 h later the 1^st^ viral suspension was removed and a second 5-ml aliquot of viral suspension was similarly treated and added to the plate till the next day. 48 h post infection cells were subjected to selection with 3 μg/ml puromycin. Generation of retroviruses and infection of MEFs was carried out as previously described [31].

### Cell Proliferation and Viability

Proliferation was monitored by measuring the A_650_ of the methylene-blue dye extracted from stained cells [32]. For viability assessment, cells were harvested and the proportion of viable cells was monitored by the trypan blue exclusion procedure using the TC10 automated cell counter (Bio Rad) and following the manufacturer’s instructions.

### Measurement of the Rate of Protein Synthesis

Cells were incubated for 1 h with 1 μM puromycin, and the extent of puromycin incorporated into the pulse labeled nascent peptides was assessed by Western blot analysis with anti-puromycin antibody.

### Polysomal Fractionation

Polysomal fractionation was carried out as previously described [31]. After centrifugation, the A260 was continuously monitored and recorded by VISIONlite (Thermo Spectronic) attached to a Genesis 10uv (Thermo Spectronic) spectrophotometer. The gradients were divided into 12 fractions and each was treated at room temperature for 15 min with 0.02% Na^+^-deoxycholate. Proteins were precipitated with 10% trichloroacetic acid (60 min at room temp.) and spun down at 14 krpm of 10 min at 4°C. Ice cold acetone was added to the protein precipitate, mixed by Vortex mixer and left on ice for 20 min. After spinning down at 14 krpm of 10 min at 4°C the pellet was dried down, and dissolved for Western blot analysis.

### In Gel Proteolysis and Mass Spectrometry Analysis

The proteins in the gel were reduced with 2.8 mM DTT (60°C for 30 min), modified with 8.8 mM iodoacetamide in 100 mM ammonium bicarbonate (in the dark, at room temperature, for 30 min) and digested in 10% acetonitrile and 10 mM ammonium bicarbonate with modified trypsin (Promega, 1:10) overnight at 37°C. The resulting tryptic peptides were resolved by reverse-phase chromatography on 0.075 X 200-mm fused silica capillaries (J&W) packed with Reprosil reversed phase material (Dr. Maisch GmbH, Germany). The peptides were eluted with linear 65 minutes gradients of 5% to 45% and 15 minutes at 95% acetonitrile with 0.1% formic acid in water at flow rates of 0.25 μl/min. Mass spectrometry was performed by an ion-trap mass spectrometer (OrbitrapXP, Thermo) in a positive mode using repetitively full MS scan followed by collision induces dissociation (CID), with multistage activation, of the 7 most dominant ion selected from the first MS scan. The mass spectrometry data was analyzed using the Protein Discoverer 1.3 (ThermoFisher inc.) using both Sequest and Mascot search engines, searching against the Human section of the Uniprot database. Identifications were filtered according to mass accuracy and 1% false discovery rate (FDR).

### Luciferase Assay

Immortalized MEFs were cotransfected with plasmids expressing Renilla or firefly luciferase using the Jetprime reagent according to the manufacturer’s (Polyplus-transfection SA, France) instruction. Harvesting the cells, preparation of the extract and measurement of the luciferase activities were performed as previously described [33].

## Results

### Akt1 Overexpression is Dominant Over rpS6 Phosphorylation Deficiency in Triggering β-Cell Hypertrophy and Enhanced Function

rpS6 phosphorylation deficient mice exhibit decreased β-cell size and impaired glucose tolerance [15]. The location of Akt1 upstream of rpS6 raised the possibility that Akt1 might induce β-cells growth, at least partly, by activation of rpS6 phosphorylation. To examine whether rpS6 and myr-Akt1 alleles interact epistatically, we crossed rpS6^P-/-^ knockin mice [15] with mice overexpressing myr-Akt1 in β-cells (Akt^tg^) [7], yielding all four possible genotypes (Fig 1A). Clearly, constitutive expression of the Akt^tg^ resulted in upregulation of the mTORC1 activity, as can be judged from the hyperphosphorylation of its downstream target, rpS6, and the dramatic increase in β-cell size, relative to that of WT cells in both 2-mo- and 12-mo-old mice (Fig 1A and S1 Fig).

Morphometric analysis confirmed the opposite effects of rpS6 phosphorylation deficiency and constitutive Akt1 activity on β-cell size (smaller β-cells in rpS6^P-/-^ mice and larger β-cells in Akt^tg^ mice, Fig 1A and 1B, as well as [6, 7, 15]). Yet, despite the fact that Akt1 resides upstream of rpS6 phosphorylation, the lack of rpS6 phosphorylation surprisingly failed to restrict β-cell size in the double mutant (compare Akt^tg^ with rpS6^P-/-^;Akt^tg^ in Fig 1A, 1B and 1C and in S3 and S4 Figs). It has previously been reported that the small-cell-size phenotype of rpS6^P-/-^ MEFs is accompanied by accelerated proliferation rate, relative to WT MEFs [15]. Likewise, the engagement of rpS6^P-/-^ β-cells in the cell cycle, as exemplified by the percentage of Ki67-positive cells, significantly exceeds that of their WT counterparts. Interestingly, β-cell replication was further increased in rpS6^P-/-^;Akt^tg^ mice (Fig 1D).

The reciprocal impact of rpS6 phosphorylation deficiency and constitutively active myr-Akt1 on β-cell size is reflected in opposite effects on glucose tolerance (Fig 1E) and glucose-stimulated insulin secretion (Fig 1F), namely impaired in rpS6^P-/-^ while improved in Akt^tg^, relative to WT. In accordance with the dominance of constitutively active myr-Akt1 over rpS6 phosphorylation deficiency on β-cell size, the deficiency of rpS6 phosphorylation failed to correct glucose tolerance or insulin secretion in Akt^tg^ mice. In fact, rpS6 deficiency further exaggerated, rather than inhibited, insulin secretion in Akt^tg^ mice (Fig 1F). Interestingly, the relative total β-cell area in rpS6^P-/-^;Akt^tg^ mice was indistinguishable from that of Akt^tg^ mice. Moreover, these genotypes displayed a 2.4- and 3.1-fold increase, relative to that of rpS6^P-/-^ and WT mice, respectively (Fig 1G). Conceivably, the apparent similar β-cell area in WT and rpS6^P-/-^ mice (Fig 1G and [15]), despite the small-cell-size phenotype of rpS6^P-/-^ β-cells, reflects an elevation in the number of the latters, due to their enhanced proliferation rate (Fig 1D). Collectively, these results indicate that constitutively active myr-Akt triggers increased β-cell mass and enhances insulin secretion in an rpS6 phosphorylation-independent fashion.

### Phosphorylation of rpS6 is Required for the Development of Akt1-Induced Insulinoma

Akt^tg^ mice, but not wild type or rpS6^P-/-^ mice, died at 10–15 months of age as previously reported [26], and this was preceded by hypoglycemia (data not shown). Pathological analysis of the dying mice disclosed the development of insulinoma in two thirds of these mice (Fig 2C), twice as many as previously reported for this genotype [26]. Strikingly, while rpS6 phosphorylation was nearly excluded from islets of 12-Mo old WT mice, it became readily detectable in insulinoma cells in aged-matched Akt^tg^ mice (Fig 2A). In order to examine whether augmented phosphorylation of rpS6 is a byproduct or, alternatively, a prerequisite for the tumorigenic process, we searched for insulinoma in rpS6^P-/-^;Akt^tg^ mice. Strikingly, none of 12 examined mice exhibited insulinoma, even at the age of 15 months (Fig 2B and 2C).

Monitoring Ki67 positive β-cells disclosed an enhanced proliferation rate of β-cells missing rpS6 phosphorylation, relative to WT cells at different time points between 1 and 12.5 months of age (Fig 2D). This observation accords with our earlier report on enhanced proliferation of rpS6^P-/-^ MEFs [15]. The extent of enhanced proliferation of Akt^tg^ β-cells in 1 or 2-Mo old mice is comparable with that previously documented for 5-weeks old mice of the same genotype [6]. However, while the proliferation of Akt^tg^ β-cells is dramatically increased in insulinoma at the age of 12 month, it is maintained nearly unchanged in rpS6^P-/-^;Akt^tg^ mice at the same age (Fig 2D and S3 Fig). It has previously been reported that the expression of p27^Kip^, a cyclin-dependent kinase inhibitor [34], is reduced in insulinomas [35]. Consistently with this report, fewer Akt^tg^ β-cells had nuclear p27. Strikingly, rpS6 phosphorylation deficiency rescued nuclear localization of p27 in rpS6^P-/-^;Akt^tg^ relative to Akt^tg^ β-cells cells (Fig 2E and S4 Fig). These findings suggest an Akt-rpS6-p27 axis that controls β-cell proliferation and insulinomas formation. Alternatively, however, p27 status might be a just a reflection and not a cause of hyperproliferation and malignant transformation in Akt^tg^ mice.

One explanation for the neutralizing effect of the mutations in rpS6 in aging mice might be phenotypic changes that lead to a general loss of the dominance of myr-Akt over rpS6 phosphorylation deficiency. However, comparing the β-cell size at different ages disproved this hypothesis, since the dominance of Akt^tg^ over rpS6 phosphorylation deficiency on the cell size phenotype was preserved in aging mice (Figs 2F, 1B and 1C and S3 Fig [see staining for insulin]).

Collectively, these results imply that the tumorigenic effect of myr-Akt in β-cells is mediated by rpS6 phosphorylation and is accompanied by augmented cell proliferation at the onset of the tumor progression, with p27 as a potential effector downstream of Akt and rpS6.

### Akt-Induced Aneuploidization of β-Cells is rpS6 Phosphorylation-Independent

**Fig 3:**
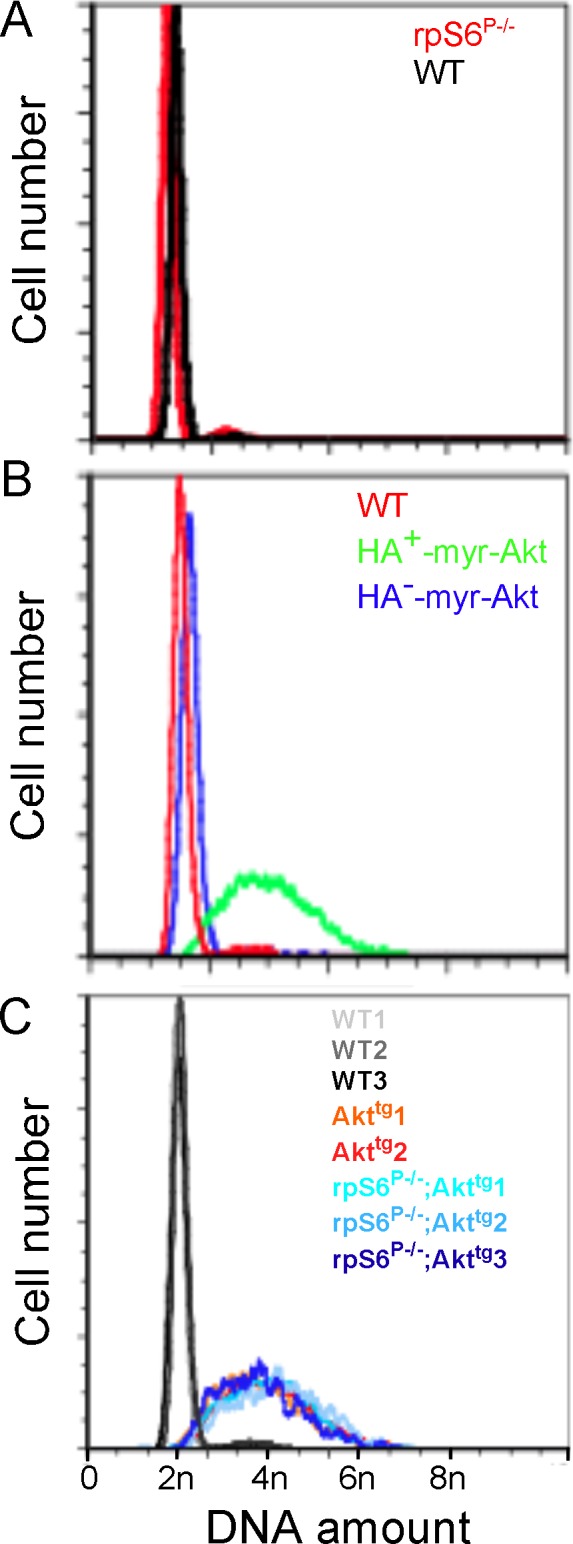
Constitutive expression of Akt induces polyploidy regardless of the rpS6 phosphorylation status. A. rpS6 deficiency does not affect ploidy in β-cells. Islets were isolated from wild-type (WT) and 2 month-old Akt^tg^ mice. Dissociated islets were fixed, permeabilized, immunostained for Insulin, HA, Ki67 and Hoechst before FACS analysis. The DNA content of 5,000 to 10,000 quiescent β-cells (insulin-positive, Ki67-negative) was measured by FACS analysis. B. 5,000 quiescent (Ki67-negative) β-cells (insulin-positive) that express myr-Akt (HA-positive) are polyploidy, whereas their HA-negative counterpart shows mostly a diploid profile as wild-type β-cells. C. rpS6 deficiency does not affect the ploidy profile of myr-Akt expressing β-cells. Islets were isolated from 2 month-old mice (three wild-type (WT), two Akt^tg^ and three rpS6^P-/-^;Akt^tg^ mice) and 5,000 to 10,000 HA-positive β-cells processed as in (A) for FACS analysis.

Developmentally programmed polyploidization occurs in multiple mammalian cell types (reviewed in [36]), including hepatocytes, which become polyploid following a failure of cytokinesis [37]. Interestingly, this inherent hepatic behavior relies on Akt, as pharmacological inhibition of Akt reduced the frequency of cytokinesis failure events in rat liver. Moreover, oncogenic Akt was shown to promote genome instability by repressing homologous recombination repair under pathologic circumstances [38]. It has been proposed that oncogenes induce DNA replication stress, and thereby genomic instability, which often leads to aneuploidy and eventually to neoplastic transformation [39]. Based on these observations, we hypothesized that constitutive expression of Akt might lead to polyploidy or aneuploidy in β-cells, potentially comprising a mechanism leading to insulinoma. We, therefore, monitored DNA content in β-cells of Akt^tg^ and rpS6^P-/-^ mice and examined how ploidy is affected by rpS6 phosphorylation deficiency. As shown in Fig 3A, dissociated islet cells from rpS6^P-/-^ and wild type mice displayed similar amounts of DNA, as assessed by FACS after Heochst staining. In contrast, β-cells from HA^+^-Akt^tg^ mice were tetraploid on average, but displayed a wide distribution in the intensity of Hoechst staining (Fig 3B), suggesting excess or lacking chromosomes in many cells. As an internal control, we took advantage of the mosaic expression of the HA-myr-Akt transgene in β-cells (expressed in most β-cells, as explained in the legend to Fig 1C and demonstrated in S5 Fig). We immunostained Akt^tg^ cells for the HA tag attached to the transgene and combined it with Hoechst staining for DNA content. HA^+^ but not HA^-^ islet cells had a higher DNA content (Fig 3B), demonstrating that constitutively active Akt drives β-cell aneuploidy cell-autonomously. Thus, β-cell aneuploidy is triggered by constitutive activity of Akt. This novel effect of Akt could theoretically contribute to the oncogenic effect of Akt in β-cells and other systems.

We then tested how rpS6 phosphorylation impacts β-cell ploidy. Strikingly, islet cells of rpS6^P-/-^;Akt^tg^ mice were aneuploid to a similar extent as cells from Akt^tg^ mice (Fig 3C). Thus, rpS6 phosphorylation is not required for Akt-induced aneuploidy. Combined with the inhibitory effects of rpS6 mutants on insulinoma development, this finding indicates that aneuploidy is not sufficient for malignant transformation of β-cells, and that deficient phosphorylation of rpS6 does not prevent insulinomas development by interfering with ploidy.

### Immortalized MEFs as an Experimental Model to Study the Antitumorigenic Effect of rpS6 Phosphorylation Deficiency

The protection against the development of Akt-induced insulinoma in rpS6^P-/-^;Akt^tg^ mice could not be explained by either restrained proliferation rate or altered ploidy of β-cells. Hence, we established an experimental model, based on immortalized WT and rpS6^P-/-^ MEFs [15] for exploring the underling mechanism.

We have previously shown that the development of precursor lesions for pancreatic ductal adenocarcinoma (PanIN lesions), in response to either chemical carcinogenesis or expression of transgenic mutant Kras, is greatly attenuated in rpS6^P-/-^ mice. Furthermore, acinar to ductal metaplasia regions in rpS6^P-/-^ mice displayed elevated DNA damage markers, specifically responding to double strand breaks [19]. Hence, protection from cancer in rpS6^P-/-^ mice was likely due to enhanced elimination of damaged cells. Hence, we initially examined the validity of our in vitro experimental system, by relying on Kras.

**Fig 4:**
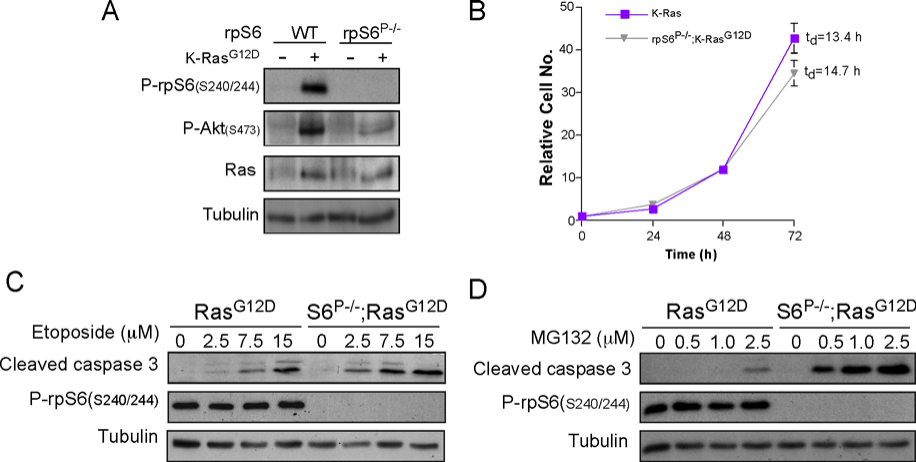
rpS6 phosphorylation deficiency renders Ras^G12D^ MEFs more sensitive to both genotoxic and proteotoxic stresses. (A) Immortalized WT, rpS6^P-/-^, Ras^G12D^ and rpS6^P-/-^;Ras^G12D^ MEFs were harvested and their cytoplasmic proteins were subjected to Western blot analysis with the indicated antibodies. (B) Ras^G12D^ and rpS6^P-/-^;Ras^G12D^ MEFs were seeded in 96-well plates at a density of 4 x10^3^ per well. Proliferation was monitored by measuring the A_650_ of the methylene-blue dye extracted from stained cells [32]. Absorbance measured 24 h after platting, was set arbitrarily at 1 and absorbance measured at later time points (average ± SEM [n = 6]) for each time point) was normalized to that value. t_d_, population-doubling time. (C) Immortalized Ras^G12D^ and rpS6^P-/-^;Ras^G12D^ MEFs were incubated with the indicated concentrations of etoposide for 24 h, harvested and their cytoplasmic proteins were subjected to Western blot analysis with the indicated antibodies. (D) Immortalized Ras^G12D^ and rpS6^P-/-^;Ras^G12D^ MEFs were incubated with the indicated concentrations of MG132 for 24 h, harvested and their cytoplasmic proteins were subjected to Western blot analysis with the indicated antibodies.

**Fig 5:**
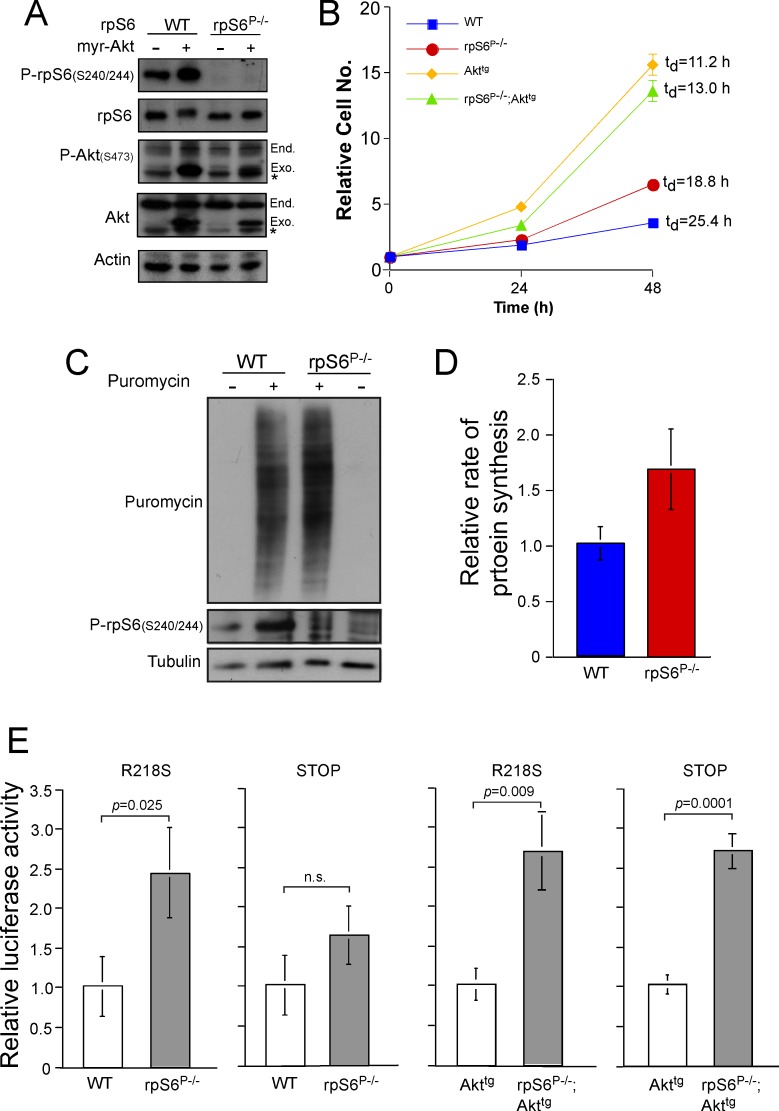
rpS6 phosphorylation deficiency increases proliferation and decreases translational fidelity in fibroblasts. (A) Immortalized WT, rpS6^P-/-^, Akt^tg^ and rpS6^P-/-^;Akt^tg^ MEFs were harvested and their cytoplasmic proteins were subjected to Western blot analysis with the indicated antibodies. (B) MEFs described in (A) were seeded in 96-well plates at a density of 4 x10^3^ per well. The proliferation of MEFs described in (A) was monitored as described in (Fig 4B). t_d_, population doubling time. (C) WT and rpS6^P-/-^ immortalized MEFs were incubated for 1 h with 1 mM puromycin, harvested and their cytoplasmic proteins were subjected to Western blot analysis with the indicated antibodies. (D) Quantification of signals obtained in 6 experiments similar to that described in (C). (E) WT, rpS6^P-/-^, Akt^tg^, and rpS6^P-/-^;Akt^tg^ MEFs were cotransfected with Renilla luciferase expression vector and any one of the following firefly luciferase expression vectors: Fluc(WT), Fluc(Stop) and Fluc(R218S). The transfection efficiency of any of the Fluc constructs was normalized to that of the Renilla luciferase. The relative Fluc activity of each mutant was normalized to Fluc (WT). The result obtained for each mutant in WT MEFs (n = 24) was arbitrarily set at 1 and the relative luciferase activity obtained for each Fluc mutant in rpS6^P-/-^ MEFs (n = 24) was normalized to that value. Similarly, results obtained for each mutant in Akt^tg^ MEFs (n = 12) were arbitrarily set at 1 and the relative luciferase activity obtained for each Fluc mutant in rpS6^P-/-^;Akt^tg^ MEFs (n = 12) was normalized to that value.

WT and rpS6^P-/-^ MEFs were infected with a retrovirus expressing Kras, bearing the same mutation as in the mouse pancreas (Ras^G12D^). The resulting cells had increased Ras expression relative to uninfected cells (Fig 4A), and had higher level of Ser473 phosphorylation in Akt, as has previously been shown [40]. Fig 4B demonstrates an enhanced rate of proliferation, as inferred from the population doubling time (t_d_), in Ras^G12D^ expressing MEFs (13.4 h), relative to that of WT MEFs (t_d_ = 25.4 h, Fig 5B).

To induce a genotoxic stress, MEFs were treated with etoposide, which prevents re-ligation of the DNA strands, through inhibition of topoisomerase II enzymes, leading to DNA breaks and consequently leads to apoptosis [41, 42]. Interestingly, when rpS6^P-/-^;Ras^G12D^ MEFs were subjected to such a treatment they exhibited enhanced sensitivity, relative to Ras^G12D^ MEFs, as exemplified by the reduced appearance of cleaved caspase 3 (Fig 4C). These results are in a good agreement with the increased DNA damage documented in PanIN lesions of rpS6^P-/-^;Ras^G12D^ vs. Ras^G12D^ mice, and support the hypothesis that absence of rpS6 phosphorylation may negate Kras-driven cancer development by enhancing tumor suppressive DNA damage [19]. This hypersensitivity is not confined just to genotoxic stress, as rpS6 phosphorylation deficiency rendered Kras-expressing MEFs more sensitive to a proteasome inhibitor, GM132, that blocks both normal protein turnover and removal of aberrant proteins (Fig 4D).

### The Nuclear Proteins Topoisomerase 2b and Psip1 Selectively Bind Unphosphorylatable rpS6

rpS6 phosphorylation deficiency exerts a wide range of effects that include reduced cell size, increased proliferation rate, reduced insulin secretion from beta cells [15], tumor suppression in pancreata expressing Akt^tg^ or Ras^G12D^ (Fig 2C and [19]), and altered apoptotic response to proteotoxic and genotoxic stressors in fibroblasts (Fig 4). To better understand these effects we searched for proteins that selectively interact with unphosphorylatable rpS6. First, we infected HEK293 cells with lentiviral vectors expressing three different rpS6-GFP chimeric proteins: pS6^5S^-GFP (rpS6 with 5 phosphorylatable serine residues, equivalent to WT), pS6^5A^-GFP (S to A replacement of all phosphorylatable serine residues, equivalent to rpS6^P-/-^ mutant) and pS6^5D^-GFP (S to D replacement of all phosphorylatable serine residues, phosphomimetic mutations). The detection of the GFP moiety within polysomal fractions indicated that all three mutants were not only expressed in the cell, but also assembled into translating ribosomes (S6 Fig). Whole cell extracts from these cells were prepared, GFP-tagged proteins were pulled down and were subjected, together with their interactors, to mass spectrometric analysis. Table 1 presents six proteins that were identified to specifically bind pS6^5A^-GFP, but not GFP alone, pS6^5S^-GFP or pS6^5D^-GFP.

**Table 1:** List of proteins that selectively interact with unphosphorylatable form of rpS6. Whole cell extract from HEK293 cells infected with pS6^[5S]^-GFP, pS6^[5A]^-GFP, pS6^[5D]^-GFP or pEGFP-N1, was subjected to GFP pull-down, and the bound proteins were size fractionated by SDS-polyacrlamide gel electrophoresis. Mass spectrometric analysis of proteins in each lane was performed as described in “material and Methods” and proteins, selectively bound to pS6^[5A]^-GFP in two independent experiments, are presented (numbers separated by slash [/] represent results obtained in each of the two individual analyses).

Gene name	Protein	Function	Location	Area^a^	Coverage^b^	No. of unique peptides^c^
**PSIP1**	PC4 and SFRS1-interacting protein 1 (LEDGF)	Repair of DNA double-strand breaks	Nucleus	1.141E6/1.705E7	5.28/17.55	3/8
**TOP2B**	Topoisomerase (DNA) II beta	DNA replication, transcription and repair	Nucleus	7.922E6/2.783E7	19.62/8.98	18/7
**SRSF4**	Serine/arginine-rich splicing factor 4	Splicing	Nucleus	2.327E6/3.816E7	7.89/11.54	3/2
**ABCD3**	ATP-binding cassette, sub-family D (ALD), member 3	Transporter (?)	Peroxysomal and mitochondrial membranes	1.584E6/9.683E6	6.83/3.95	3/2
**NDUA9**	NADH dehydrogenase (ubiquinone) 1 alpha subcomplex 9	Accessory subunit of Complex I	Mitochondria	5.180E5/8.213E6	8.49/9.28	3/3
**SYQ**	Glutaminyl-tRNA synthetase	Translation	Cytoplasm	1.596E6/5.299E6	6.32/5.42	4/3
**PSIP1**	PC4 and SFRS1-interacting protein 1 (LEDGF)	Repair of DNA double-strand breaks	Nucleus	1.141E6/1.705E7	5.28/17.55	3/8
**TOP2B**	Topoisomerase (DNA) II beta	DNA replication, transcription and repair	Nucleus	7.922E6/2.783E7	19.62/8.98	18/7
**SRSF4**	Serine/arginine-rich splicing factor 4	Splicing	Nucleus	2.327E6/3.816E7	7.89/11.54	3/2
**ABCD3**	ATP-binding cassette, sub-family D (ALD), member 3	Transporter (?)	Peroxysomal and mitochondrial membranes	1.584E6/9.683E6	6.83/3.95	3/2
**NDUA9**	NADH dehydrogenase (ubiquinone) 1 alpha subcomplex 9	Accessory subunit of Complex I	Mitochondria	5.180E5/8.213E6	8.49/9.28	3/3
**SYQ**	Glutaminyl-tRNA synthetase	Translation	Cytoplasm	1.596E6/5.299E6	6.32/5.42	4/3

^a^ Area—displays the average area of the three unique peptides with the largest peak area, based on extracted ion currents (XICs).

^b^ Coverage—displays the percentage of the protein sequence covered by identified peptides.

^c^ No. of unique peptides—Displays the number of peptide sequences unique to a protein group.

Surprisingly, three of the pS6^5A^-GFP-binding proteins, Psip1 (PC4 and SFRS1-interacting protein 1, also known as LEDGF), TOP2B (topoisomerase IIβ) and SRSF1 (Serine/arginine-rich splicing factor 4), are nuclear. It has previously been shown that genotoxicity of etoposide is TOP2B-dependent [43, 44] and that Psip1 is involved in DNA repair, among its other functions [45]. Clearly, even though these interacting partners of unphosphorylated rpS6 might provide a link between rpS6 and DNA damage response, additional experiments will be required for establishing such a functional relationship.

### rpS6 Phosphorylation Deficiency Accelerates Cell Proliferation and Decreases Translational Fidelity *In Vitro*

The consistency of the results obtained for Kras *in vitro* with those obtained *in vivo*, prompted us to use immortalized MEFs to approach the mechanism underlying the antitumorigenic effect of rpS6 phosphorylation in the presence of constitutively active Akt1. WT and rpS^P-/-^ MEFs were infected with a lentivirus expressing myr-Akt (Fig 5A). A higher rate of proliferation was observed in rpS6^P-/-^, relative to WT MEFs, in a close agreement with that observed in β-cells of these genotypes (compare Fig 5B with Fig 2D). Moreover, the proliferation of rpS6^P-/-^;Akt^tg^ MEFs was attenuated relative to Akt^tg^ immortalized MEFs (Fig 5B), although the effect was markedly less than that observed for β-cells from 12-mo-old mice (compare Fig 5B with Fig 2D). This difference might reflect the fact that both Akt^tg^ and rpS6^P-/-^;Akt^tg^ MEFs are immortalized, whereas in 12-mo-old mice only the Akt^tg^, but not rpS6^P-/-^;Akt^tg^, β-cells are transformed.

It has been previously shown that increased rate of protein synthesis leads to decreased translation fidelity [46]. Since primary rpS6^P-/-^ MEFs have higher rates of both proliferation and protein synthesis [15], we hypothesized that the fidelity of the translation in this genotype might be impaired. Hence, we monitored the rate of protein synthesis in immortal WT and rpS6^P-/-^ MEFs by pulse labeling of nascent peptide chains with puromycin. Although the difference between the two genotypes was statistically insignificant, rpS6^P-/-^ MEFs displayed a tendency toward a higher rate of protein synthesis, which is consistent with the faster proliferation (Fig 5C and 5D).

To test if this tendency might correlate with decrease translation fidelity, we transfected cells with two luciferase reporters: a) Fluc(R218S), in which the arginine at the active-site (position 218) was mutated into serine that renders the resultant mutant protein devoid of enzymatic activity [46], and b) Fluc(Stop) that has leucine at position 210 replaced with a stop codon, which leads to the synthesis of a truncated and enzymatically inactive protein product [46]. Fig 5E shows that rpS6 phosphorylation deficiency significantly reduced translation fidelity, as judged by the increased luciferase activity if transfected with Fluc(R218S), and to a much lesser extent if transfected with Fluc(Stop). The reason for the apparent difference between the two reporters is presently unclear. Nevertheless, rpS6 phosphorylation deficiency induced a 2.6-fold reduction in the translation fidelity in rpS6^P-/-^;Akt^tg^, relative to Akt^tg^ MEFs, regardless of the luciferase mutant used (Fig 5E). These results indicate that while rpS6 phosphorylation deficiency increases overall protein synthesis, it compromises translation fidelity. Reduced fidelity of translation suggests a mechanism for how rpS6 phosphorylation deficiency may interfere with tumorigenesis.

### rpS6 Phosphorylation Deficiency Confers Resistance to Proteotoxic, or Genotoxic Stresses in Akt1 Expressing MEFs

**Fig 6:**
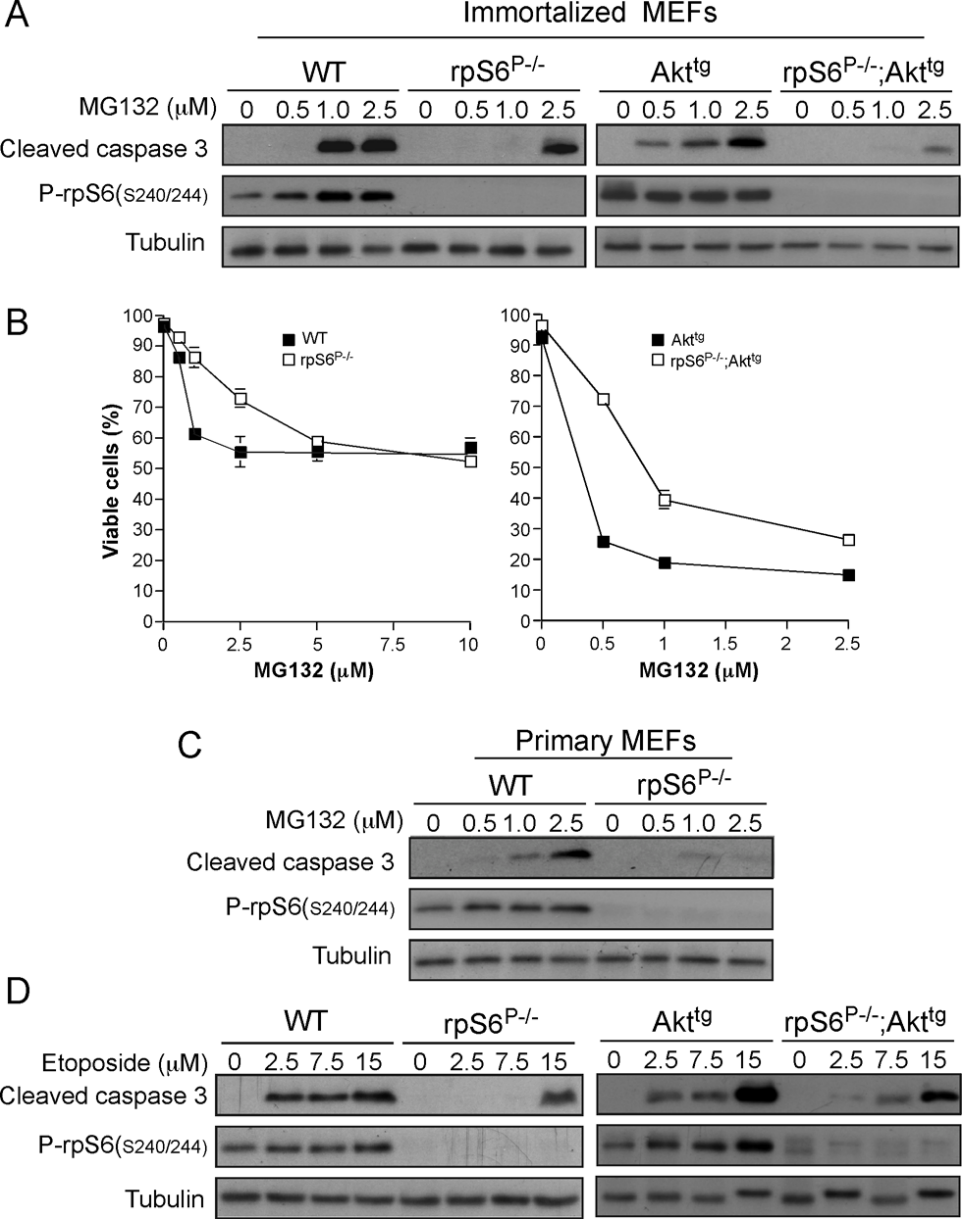
rpS6 phosphorylation deficiency renders WT or Akt^tg^ MEFs more resistant to a proteotoxic and genotoxic stress. (A) Immortalized WT, rpS6^P-/-^, Akt^tg^, and rpS6^P-/-^;Akt^tg^ MEFs were incubated with the indicated concentrations of MG132 for 16 h, harvested and their cytoplasmic proteins were subjected to Western blot analysis with the indicated antibodies. (B) Viability of cells described in (A) was assessed by the Trypan blue exclusion assay, as described in Material and Methods. The percentage of viable cells (average of six wells for each time point) is presented as mean ± SEM (the size of the symbol for most measurements was larger than the respective SEM). (C) Primary WT and rpS6^P-/-^ MEFs (passage 3) were analyzed as described in (A). (D) Immortalized WT, rpS6^P-/-^, Akt^tg^, and rpS6^P-/-^Akt^tg^ MEFs were incubated with the indicated concentrations of etoposide for 24 h, harvested and their cytoplasmic proteins were subjected to Western blot analysis with the indicated antibodies.

One consequence of the lower translation fidelity in rpS6^P-/-^ and rpS6^P-/-^;Akt^tg^ MEFs might be a higher sensitivity to proteotoxic stress, as has previously been shown for cells with excessive protein synthesis [46]. To examine this possibility, cells were treated with MG132. Unexpectedly and in a contrast to data obtained with rpS6^P-/-^;Ras^G12D^, rpS6 phosphorylation deficiency diminished MG132-induced apoptosis in both rpS6^P-/-^ and rpS6^P-/-^;Akt^tg^ MEFs (Fig 6A). This seemingly protective role of rpS6 phosphorylation deficiency is also reflected in the survival of the different genotypes under this stress, as assessed by trypan blue exclusion (Fig 6B). Notably, this behavior is not an artifact of the immortalized cells, as primary rpS6^P-/-^ MEFs demonstrated a similar resistance to MG132, relative to their WT counterparts (Fig 6C).

Next, we examined the sensitivity of rpS6^P-/-^ and rpS6^P-/-^;Akt^tg^ MEFs to genotoxic stress. As with MG132 treatment, rpS6 phosphorylation deficiency surprisingly rendered wild type as well as Akt^tg^ MEFs more resistant to the etoposide-induced apoptosis (Fig 6D). Thus, fibroblasts became more resistant to both genotoxic and proteotoxic stress in the absence of rpS6 phosphorylation, regardless of the expression of active Akt.

Collectively, these data support the notion that the phosphorylation status of rpS6 affects cellular sensitivity to stress in a context-dependent manner, i.e. rpS6 phosphorylation appears to render Kras-expressing cells more resistant to stress, while it renders Akt-expressing cells more sensitive to stress.

### rpS6 Phosphorylation Deficiency Exerts no Effect on the Autpohagic Response of MEFs Expressing Akt1

**Fig 7:**
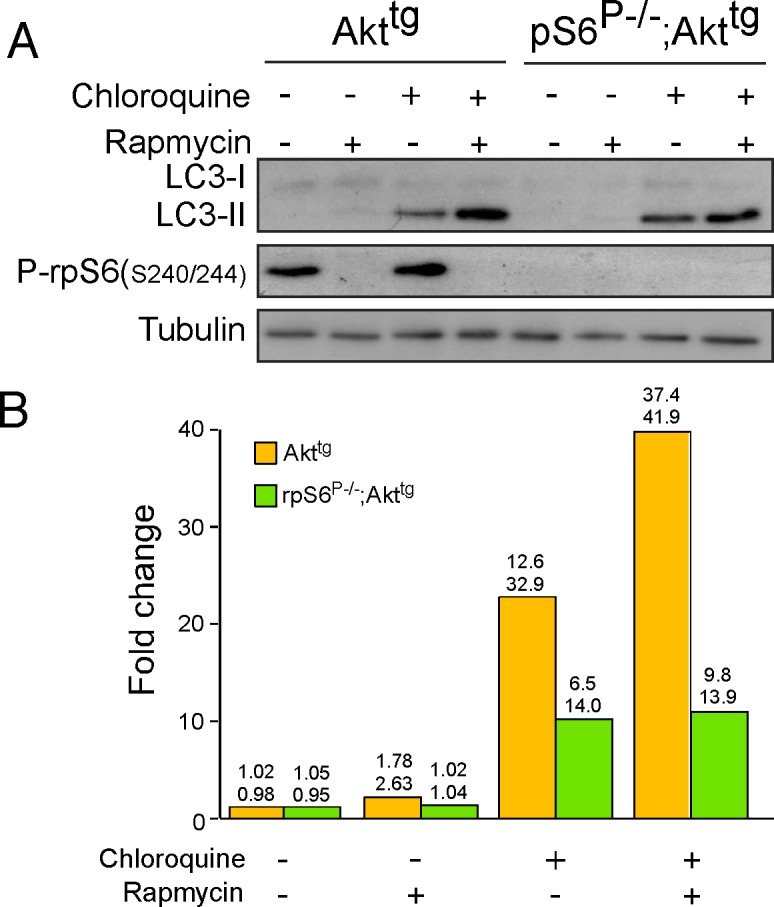
The phosphorylation status of rpS6 plays no role in autophagy. (A) Immortalized Akt^tg^ and rpS6^P-/-^;Akt^tg^, MEFs were either untreated, incubated with 20 nM rapamycin, 50 μM chloroquine or both for 16 h. Cells were harvested and their cytoplasmic proteins were subjected to Western blot analysis with the indicated antibodies. (B) The signals of LC3-II, obtained in Western blot analyses of two independent experiments, as described in (A), were quantified and normalized to that tubulin. The fold change in the expression of LC3-II upon addition of rapamycin, chloroquine or both, was related to that of untreated cells, which was arbitrarily set at one.

Autophagy impairs early cancer development, while facilitating advanced tumor progression, by serving a dual function during tumorigenesis. Thus, on the one hand its homeostatic role enables lysosomal digestion and recycling of cellular contents, and thereby limits genome-damaging events that would otherwise favor tumor initiation. On the other hand, however, the ability of autophagy to help cells mitigate stress facilitates advanced tumor progression [47]. To test whether the phosphorylation status of rpS6 might affect autophagy we treated cells with rapamycin in the presence or absence of chloroquine. Rapamycin mimics the effect of amino acid starvation by inhibiting mTORC1 and thereby is expected to induce autophagy [48], even if its effect is not always readily detectable [49]. Chloroquine inhibits the fusion of autophagy vacuoles with the lysosome [50] and thereby enables their accumulation and the detection of their marker, LC3-II [51]. The results of this experiment demonstrate that autophagy not only was not enhanced in rpS6^P-/-^;Akt^tg^ MEFs relative to Akt^tg^ MEFs, it might even have been suppressed (Fig 7).

It should be pointed out, however, that the apparent resistance of rpS6-phophorylation deficient Akt^tg^ MEFs to proteotoxic, genotoxic and autophagic stresses does not necessarily reflect the behavior of rpS6^P-/-^;Akt^tg^ β-cells within the pancreas. Evidently, novel experimental systems need to be designed in order to establish these issues *in vivo*.

## Discussion

Aberrant mTOR signaling in tumors is due to either loss of function of upstream tumor suppressor proteins, like PTEN and TSC, or activating mutations within oncogenes that feed into the mTOR pathway. Hyperactivation of the PI3K-Akt pathway is a feature of a large majority of cancer cell types [52]. Indeed, amplification, overexpression, and activation of Akt occur at high frequency in a number of human cancers [53, 54].

It has previously been shown that rpS6 phosphorylation is critical for Kras-induced PanIN lesion formation in the exocrine pancreas and for full-blown pancreatic ductal adenocarcinoma induced by a carcinogen [19]. The present report expands the reliance on rpS6 phosphorylation to tumor development in the endocrine pancreas. It should be noted, however, that this dependence does not reflect a general mechanism, but rather a tissue specific phenomenon. Thus, Akt^T^ mice, in which a constitutively active Akt2 is expressed in immature T cells, develop spontaneous thymic lymphomas, which cannot be prevented in rpS6^P-/-^;Akt^tg^ MEFs double mutant mice. It appears, therefore, that rpS6 is dispensable for transformation downstream of oncogenic Akt signaling in the thymus [55].

Activated Akt modulates the function of numerous substrates, which regulate many cell processes [56]. Conceivably, the expression of constitutively active Akt1 could induce insulinoma via activation of the mTORC1 pathway and thereby its downstream effectors, S6K1 and rpS6 phosphorylation in β-cell ([26] and the present report). The present study demonstrates a dominance of constitutively active Akt over rpS6 phosphorylation deficiency in regulating β-cell size, total β-cell area, glucose homeostasis and ploidy (Figs 1, 2 and 3).

The ability of Akt to increase cell size independently of its downstream effector rpS6 is not unprecedented. Exercise, pressure overload or forced activation of the PI3K/Akt pathway induce cardiac hypertrophy that correlates with elevated S6K1 activity and S6 phosphorylation ([57] and references therein). However, deletion of S6K1 and S6K2 and thereby elimination of rpS6 phosphorylation had no impact on the development of pathological, physiological, or genetically induced cardiac hypertrophy [57]. These results imply that cardiac hypertrophy, even when induced in a PI3K/Akt-dependent manner, does not rely on S6K activation or rpS6 phosphorylation, consistently with our findings in β-cells.

One plausible model, which might explain this seemingly paradoxical observation, is that Akt exerts its stimulatory role on cell size through mutually exclusive pathways: one is rpS6 phosphorylation-dependent and the second is rpS6 phosphorylation-sensitive. Hence, deficiency of S6K activity or rpS6 phosphorylation alleviates repression of the alternative pathway, and thereby enables Akt-induced increase in cell size in an rpS6 phosphorylation-independent fashion. Contrarily, data presented here clearly support the notion that Akt appears to fully rely on rpS6 phosphorylation when operating as an oncogene.

Ribosomopathies are a set of genetic diseases that are collectively characterized by ribosome hypofunction at the cellular level. Patients initially show hypoproliferative disorders like anemias and bone marrow failure, but tend to develop cancer at a letter stage ([58] and references therein). We show here that rpS6 knockin mutant β-cells are protected from the development of Akt-induced insulinoma, in a manner that resembles that observed in early human ribosomopathies (i.e. suppressed proliferation). Moreover, we demonstrate here for the first time that the rpS6 phosphorylation deficiency is associated with about 2.6-fold decrease in translation fidelity in cells expressing a constitutively active Akt (Fig 5E). It should be noted, however, that we have not establish a causal relationship between the reduced translation fidelity and the anti-tumorigenic effect of the mutant rpS6. Likewise, we could not exclude the possibility that the reduced translation fidelity reflects the five serine to alanine substitutions in rpS6^P-/-^ MEFs, rather than phosphorylation deficiency of this ribosomal protein.

Many of the phenotypic manifestations of mouse deficient of S6K1 are recapitulated in the rpS6 knockin mouse. Thus both these mutants exhibit: a) small β-cell size phenotype that is accompanied by hypoinsulinemia and impaired glucose homeostasis (Fig 1E and 1F and references [12, 15]); b) small myoblasts and reduced muscle mass [16, 59]; and c) blunted compensatory renal hypertrophy following contralateral nephrectomy [18, 60]. Clearly, based on these observations it is tempting to argue that this phenotypic similarity reflects the fact that rpS6 phosphorylation is an important S6K1 effector. However, this explanation is inconsistent with the observation that rpS6 is still fully phosphorylated in S6K1 deficient mouse, due to the compensatory activity of S6K2 [26, 59, 61]. It appears, therefore, that rpS6 phosphorylation deficiency restrains the development of insulinoma by a mechanism that is distinct from that operated in S6K1 knockout mice.

rpS6 phosphorylation has been proposed to reduce Kras-induced DNA damage in acinar cells and in acinar-to-ductal metaplasia, and consequently attenuates p53-mediated tumor suppression [19]. Here we show that rpS6 phosphorylation deficiency renders Ras^G12D^ MEFs considerably more sensitive to both proteotoxic and genotoxic stresses than Ras^G12D^ MEFs expressing a phosphorylatable rpS6 (Fig 4). Moreover, pull-down experiments have demonstrated a preferential binding of unphosphorylatable (rpS6P^5A^-GTP) rpS6 chimeric protein to Psip1, a nuclear component of the DNA repair machinery [45], but not of the phosphorylatable (rpS6P^5S^-GTP) or phosphomimetic (rpS6P^5D^-GTP) proteins. Collectively, these observations support a model that the oncogenic activity of K-Ras results in increased DNA damage, whose repair is impeded by rpS6^P-/—^mediated interference and consequently cells are removed by apoptosis and thereby the tumorigenic process is blocked.

Surprisingly, rpS6 phosphorylation deficiency renders WT or Akt^tg^ MEFs, unlike Ras^G12D^ MEFs, more resistant to both proteotoxic and genotoxic stresses than rpS6^P-/-^ or rpS6^P-/-^;Akt^tg^ MEFs (Figs 4 and 6). These results suggest that Akt1 and Kras exert their oncogenic properties through distinct mechanisms, even though both show addiction to rpS6 phosphorylation. Notably, the expression of both oncogenic Ras and Akt induces DNA damage, consequently both Ras- and Akt-induced cancer cells mitigate DNA damage to a level that does not impair their proliferation (reviewed in [62, 63]).

Given that mTORC1 is activated in more than 50% of human cancers [64], there has been much interest in using rapamycin and its analogs (rapalogs) to treat tumors. However, rapalogs have had limited success as single-agent cancer therapies in hundreds of clinical trials to date [65]. One reason for this resistance is that downregulation of mTORC1, and consequently of S6K1, relieves the feedback inhibition exerted by the latter on IRS-1, and thereby upregulates the PI3K-Akt signaling [66]. Hence, development of agents to specifically target rpS6 phosphorylation, rather than S6K activity, in combination with drugs aimed at upstream targets, might be much more beneficial for treating tumors that display addiction to this modification.

Finally, this study evokes an intriguing question regarding the mechanism(s) underlying the role of rpS6 phosphorylation in regulating processes as diverse as cell growth, glucose homeostasis and pro-oncogenic activity, particularly given that rpS6 is primarily a structural protein of the ribosome. Several explanations can be proposed to account for these unique physiological functions of rpS6 phosphorylation: (a) the phosphorylation of rpS6 within, or outside the ribosome affects the translation efficiency of specific mRNAs, has yet to be identified; (b) rpS6 might be one of the many bifunctional ribosomal proteins, that can carry out extraribosomal tasks often unrelated to the mechanics of protein synthesis [67]; and (c) phosphorylated rpS6 might not affect protein synthesis, but instead interact with cellular protein(s), which consequently becomes active or inactive, and thus affects the cell physiology. Indeed, rpS6^P-/-^ mice show altered transcription of the ribosome biogenesis program [68]. Moreover, we show here that several extraribosomal proteins coimmunoprecipitated with rpS6, suggesting an *in vivo* interaction, either directly or indirectly, with these proteins.

## Supporting Information

S1 Fig Representative images of β-cells stained for Phospho-rpS6. Pancreatic sections for WT, rpS6^P-/-^, Akt^tg^ and Akt^tg^; rpS6^P-/-^ 10 to 15-month old mice were immunostained for phospho-rpS6 (green), Pdx1 (red), DNA (blue). All images are set to the same scale.
(TIF)

S2 Fig Representative images of β-cells stained for Ki67. Pancreatic sections for WT, rpS6^P-/-^, Akt^tg^ and Akt^tg^; rpS6^P-/-^ 2-month old mice were immunostained for insulin (green), Ki67 (red), and DNA (blue). All images are set to the same scale.
(TIF)

S3 Fig Representative images of β-cells stained for Ki67. Pancreatic sections for WT, rpS6^P-/-^, Akt^tg^ and Akt^tg^; rpS6^P-/-^ 10 to 15-month old mice were immunostained for insulin (green), Ki67 (red), and DNA (blue). All images are set to the same scale.
(TIF)

S4 Fig Representative images of β-cells stained for p27. Pancreatic sections for WT, rpS6^P-/-^, Akt^tg^ and Akt^tg^; rpS6^P-/-^ 10 to 15-month old mice were immunostained for insulin (green), p27 (red), and DNA (blue). All images are set to the same scale.
(TIF)

S5 Fig Representative images of β-cells stained for HA. Pancreatic sections for WT, rpS6^P-/-^, Akt^tg^ and Akt^tg^; rpS6^P-/-^ 10 to 15-month old mice were immunostained for HA. All images are set to the same scale. Note, a dashed line marks the boundaries of the islets in images of sections from WT and rpS6^P-/-^ pancreata.
(TIF)

S6 Fig Recombinant rpS6-GFP is assembled into translating ribosomes regardless of its phosphorylation status. HEK293 cells were infected with lentivirus expressing rpS6^(5S)^-GFP, rpS6^(5A)^-GFP, or rpS6^(5D)^-GFP fusion proteins or were uninfected (u.i.). Cells were harvested and their cytoplasmic extracts were size fractionated by centrifugation through sucrose gradient. The tube content was collected from the bottom, and the absorbance at 260 nm was recorded (upper panels). The vertical dashed line separates the polysomal fractions (1 to 8) from the subpolysomal fractions (9 to 12). Proteins from the indicated fractions were subjected to Western blot analysis with the indicated antibodies (lower panels).
(TIF)
