# Correction: Phosphorylated Ribosomal Protein S6 Is Required for Akt-Driven Hyperplasia and Malignant Transformation, but Not for Hypertrophy, Aneuploidy and Hyperfunction of Pancreatic β-Cells

**DOI:** 10.1371/journal.pone.0155281

**Published:** 2016-05-04

**Authors:** The PLOS ONE Staff

The first author’s name appears incorrectly in the article citation. The correct citation is: Wittenberg Dreazen A, Azar S, Klochendler A, Stolovich-Rain M, Avraham S, Birnbaum L, et al. (2016) Phosphorylated Ribosomal Protein S6 Is Required for Akt-Driven Hyperplasia and Malignant Transformation, but Not for Hypertrophy, Aneuploidy and Hyperfunction of Pancreatic β-Cells. PLoS ONE 11(2): e0149995. doi:10.1371/journal.pone.0149995.

Table 1 appears incorrectly in the published article. The last six rows are duplicates of the six above them. Please see the correct Table 1 and its caption below. The publisher apologizes for the error.

**Table 1:** List of proteins that selectively interact with unphosphorylatable form of rpS6. Whole cell extract from HEK293 cells infected with pS6^[5S]^-GFP, pS6^[5A]^-GFP, pS6^[5D]^-GFP or pEGFP-N1, was subjected to GFP pull-down, and the bound proteins were size fractionated by SDS-polyacrlamide gel electrophoresis. Mass spectrometric analysis of proteins in each lane was performed as described in “material and Methods” and proteins, selectively bound to pS6^[5A]^-GFP in two independent experiments, are presented (numbers separated by slash [/] represent results obtained in each of the two individual analyses).

Gene name	Protein	Function	Location	Area^a^	Coverage^b^	No. of unique peptides^c^
**PSIP1**	PC4 and SFRS1-interacting protein 1 (LEDGF)	Repair of DNA double-strand breaks	Nucleus	1.141E6/1.705E7	5.28/17.55	3/8
**TOP2B**	Topoisomerase (DNA) II beta	DNA replication, transcription and repair	Nucleus	7.922E6/2.783E7	19.62/8.98	18/7
**SRSF4**	Serine/arginine-rich splicing factor 4	Splicing	Nucleus	2.327E6/3.816E7	7.89/11.54	3/2
**ABCD3**	ATP-binding cassette, sub-family D (ALD), member 3	Transporter (?)	Peroxysomal and mitochondrial membranes	1.584E6/9.683E6	6.83/3.95	3/2
**NDUA9**	NADH dehydrogenase (ubiquinone) 1 alpha subcomplex 9	Accessory subunit of Complex I	Mitochondria	5.180E5/8.213E6	8.49/9.28	3/3
**SYQ**	Glutaminyl-tRNA synthetase	Translation	Cytoplasm	1.596E6/5.299E6	6.32/5.42	4/3

^a^ Area—displays the average area of the three unique peptides with the largest peak area, based on extracted ion currents (XICs).

^b^ Coverage—displays the percentage of the protein sequence covered by identified peptides.

^c^ No. of unique peptides—Displays the number of peptide sequences unique to a protein group.
