# Supplementary figures and images for: Phosphorylated Ribosomal Protein S6 Is Required for Akt-Driven Hyperplasia and Malignant Transformation, but Not for Hypertrophy, Aneuploidy and Hyperfunction of Pancreatic β-Cells
Source: PLoS One. 2016 Feb 26;11(2):e0149995. doi: 10.1371/journal.pone.0149995 (PMC4769037; doi:10.1371/journal.pone.0149995)

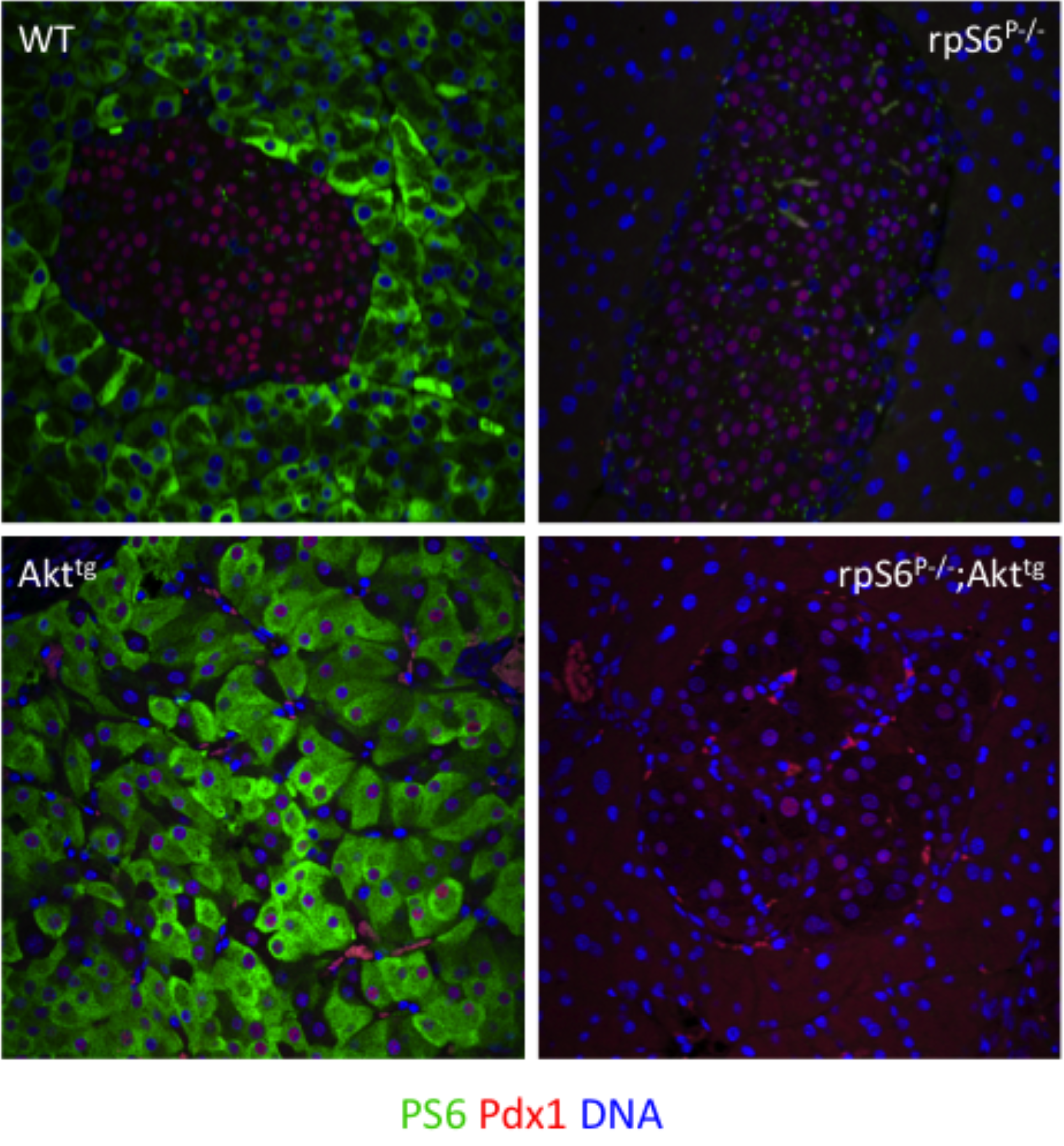

Supplement: S1 Fig — Pancreatic sections for WT, rpS6P-/-, Akttg and Akttg; rpS6P-/- 10 to 15-month old mice were immunostained for phospho-rpS6 (green), Pdx1 (red), DNA (blue). All images are set to the same scale. (TIF) [file pone.0149995.s001.tif]

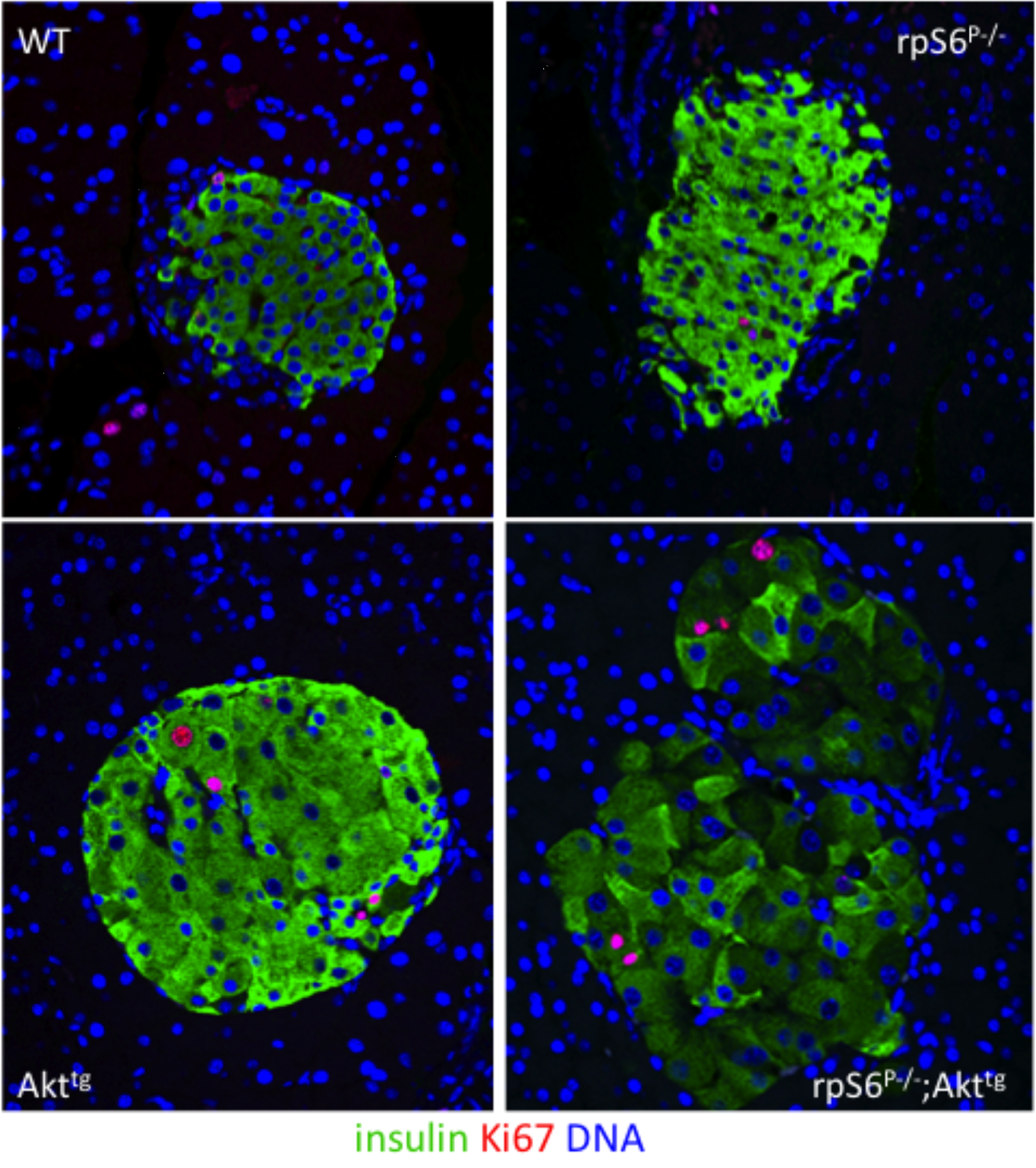

Supplement: S2 Fig — Pancreatic sections for WT, rpS6P-/-, Akttg and Akttg; rpS6P-/- 2-month old mice were immunostained for insulin (green), Ki67 (red), and DNA (blue). All images are set to the same scale. (TIF) [file pone.0149995.s002.tif]

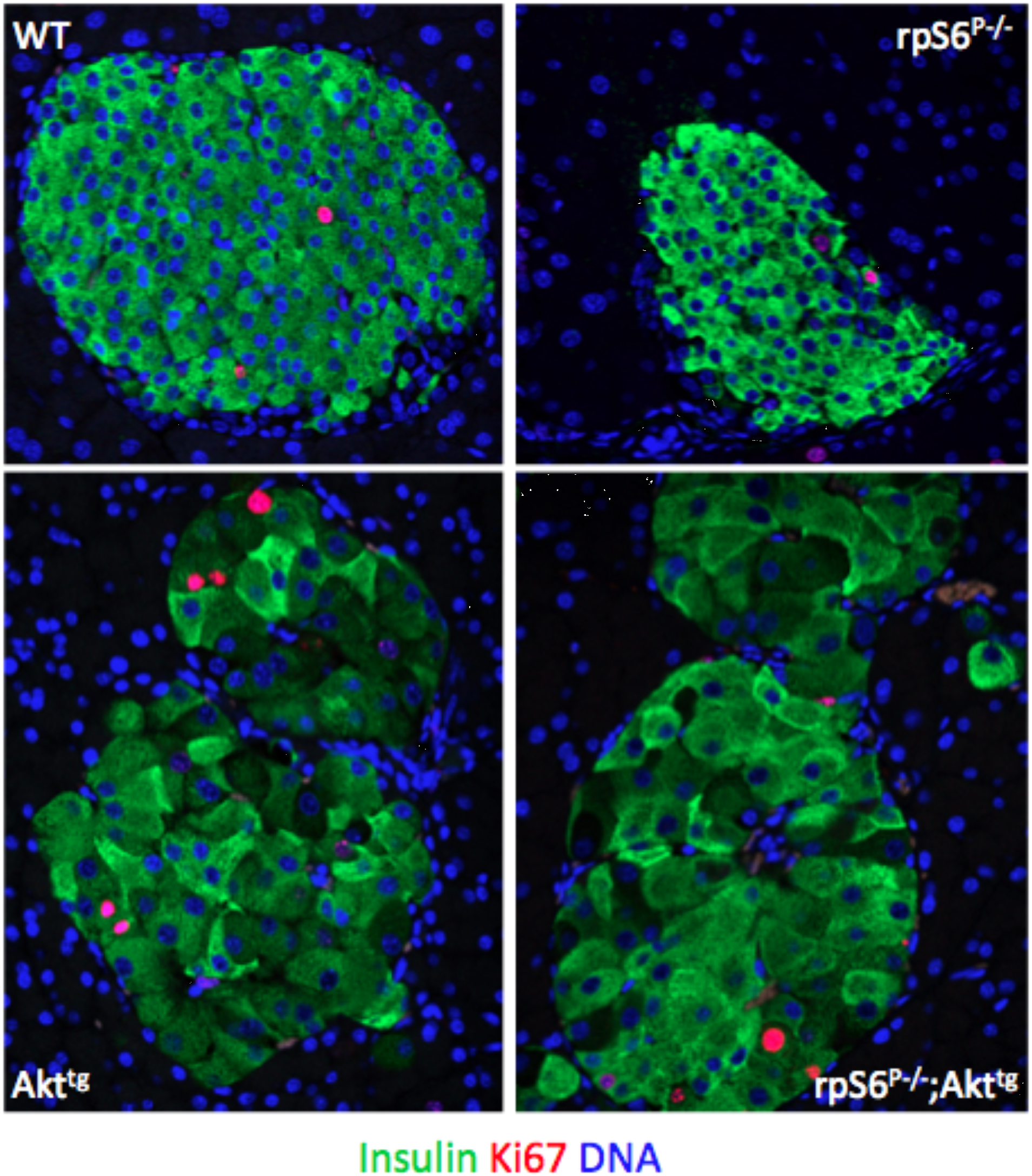

Supplement: S3 Fig — Pancreatic sections for WT, rpS6P-/-, Akttg and Akttg; rpS6P-/- 10 to 15-month old mice were immunostained for insulin (green), Ki67 (red), and DNA (blue). All images are set to the same scale. (TIF) [file pone.0149995.s003.tif]

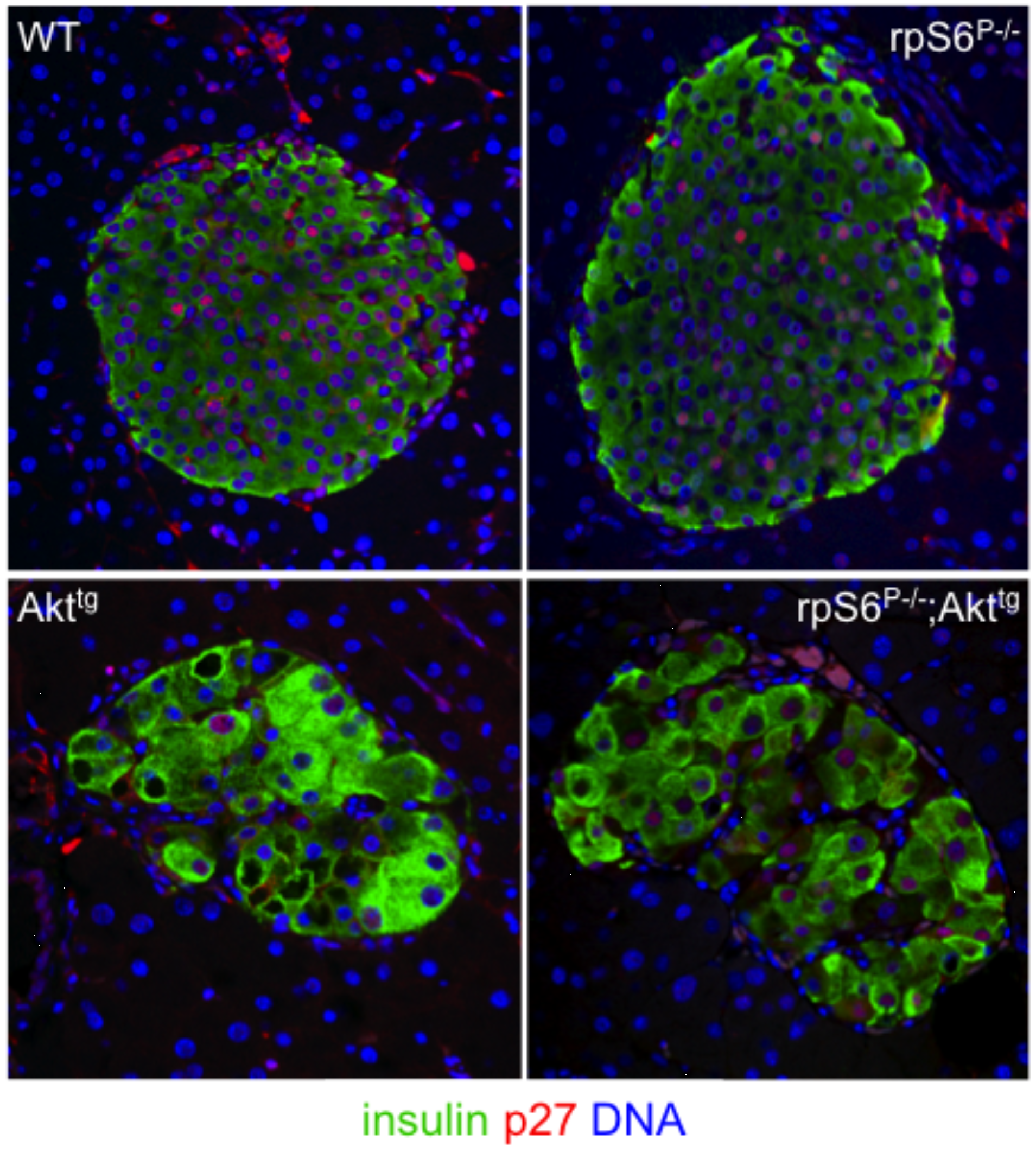

Supplement: S4 Fig — Pancreatic sections for WT, rpS6P-/-, Akttg and Akttg; rpS6P-/- 10 to 15-month old mice were immunostained for insulin (green), p27 (red), and DNA (blue). All images are set to the same scale. (TIF) [file pone.0149995.s004.tif]

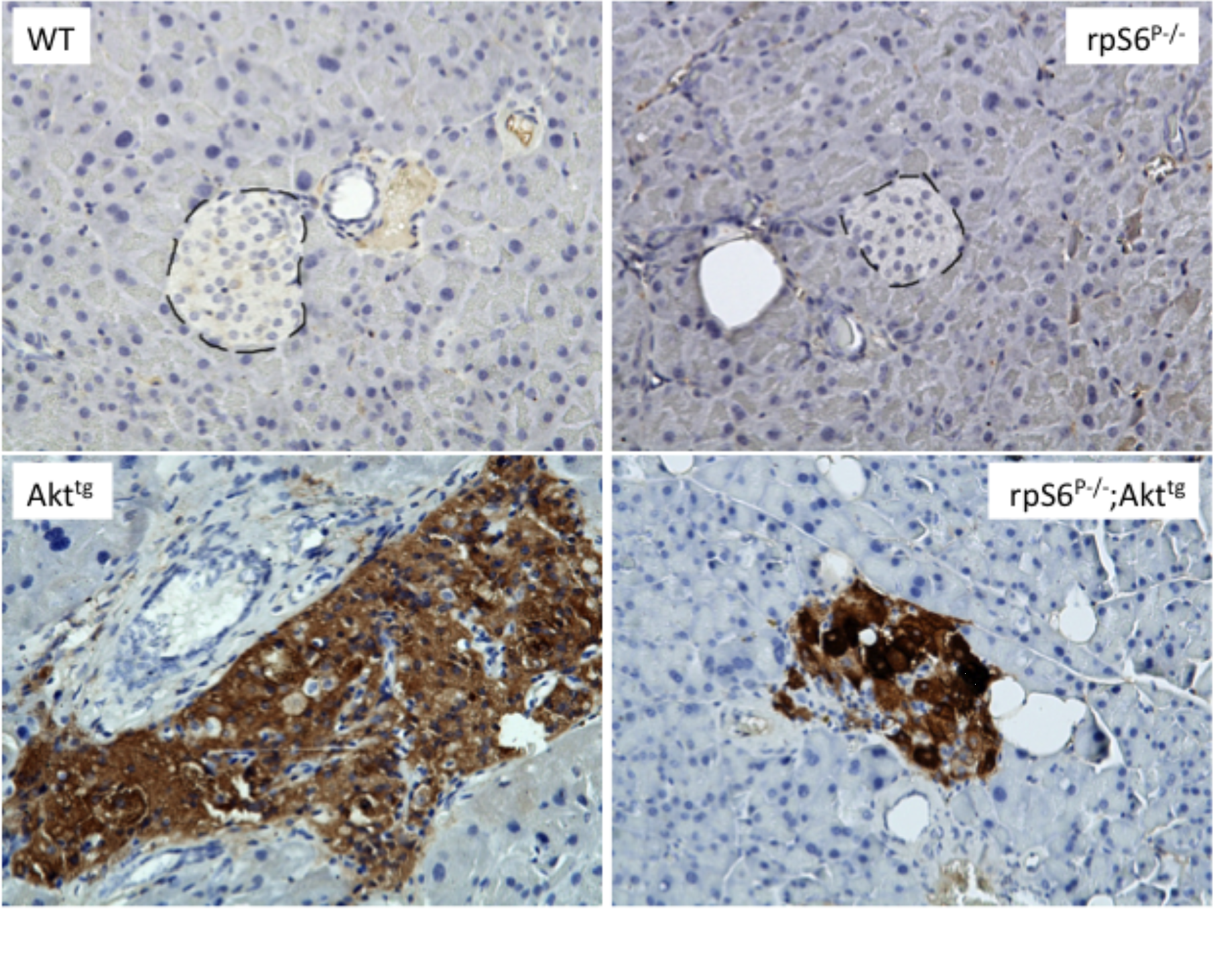

Supplement: S5 Fig — Pancreatic sections for WT, rpS6P-/-, Akttg and Akttg; rpS6P-/- 10 to 15-month old mice were immunostained for HA. All images are set to the same scale. Note, a dashed line marks the boundaries of the islets in images of sections from WT and rpS6P-/- pancreata. (TIF) [file pone.0149995.s005.tif]

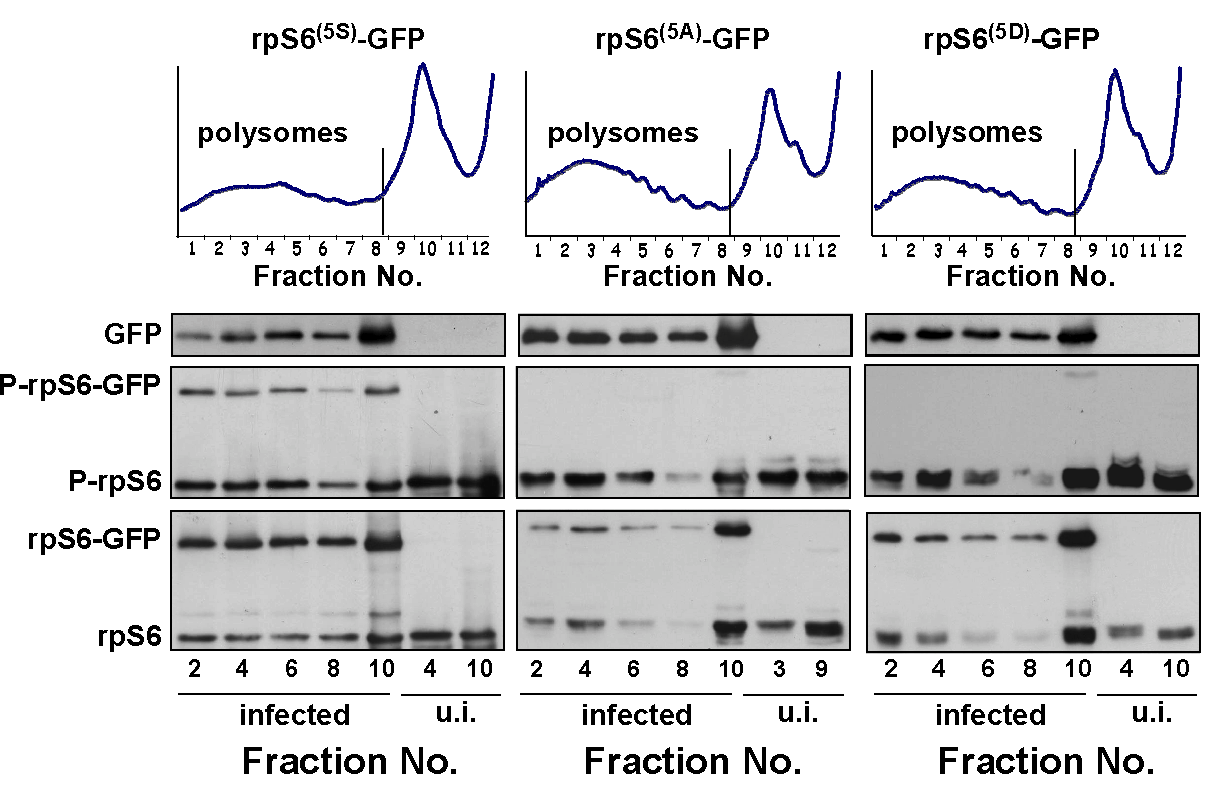

Supplement: S6 Fig — HEK293 cells were infected with lentivirus expressing rpS6(5S)-GFP, rpS6(5A)-GFP, or rpS6(5D)-GFP fusion proteins or were uninfected (u.i.). Cells were harvested and their cytoplasmic extracts were size fractionated by centrifugation through sucrose gradient. The tube content was collected from the bottom, and the absorbance at 260 nm was recorded (upper panels). The vertical dashed line separates the polysomal fractions (1 to 8) from the subpolysomal fractions (9 to 12). Proteins from the indicated fractions were subjected to Western blot analysis with the indicated antibodies (lower panels). (TIF) [file pone.0149995.s006.tif]
